# Irpexoates A–D, Four Triterpenoids with Malonyl Modifications from the Fruiting Bodies of the Medicinal Fungus *Irpex lacteus*

**DOI:** 10.1007/s13659-018-0160-3

**Published:** 2018-03-28

**Authors:** Yang Tang, Zhen-Zhu Zhao, Zheng-Hui Li, Tao Feng, He-Ping Chen, Ji-Kai Liu

**Affiliations:** 1 State Key Laboratory of Phytochemistry and Plant Resources in West China, Kunming Institute of Botany, Chinese Academy of Sciences, Kunming, 650201 People’s Republic of China; 2 School of Pharmaceutical Sciences, South-Central University for Nationalities, Wuhan, 430074 People’s Republic of China; 3 University of Chinese Academy of Sciences, Beijing, 100049 People’s Republic of China

**Keywords:** *Irpex lacteus*, Meruliaceae, Eburicane, Malonyl, Cytotoxicity

## Abstract

**Abstract:**

Four eburicane-type triterpenoids with malonyl modifications, namely irpexoates A–D (**1**–**4**), were isolated from the fruiting bodies of the medicinal fungus *Irpex lacteus*. The structures of the new compounds were established by extensive spectroscopic methods, including 1D and 2D NMR, HRESIMS spectroscopic analysis. Irpexoate B (**2**) displayed weak cytotoxicity against four human cancer cell lines (A-549, SMMC-7721, MCF-7, SW480) with IC_50_ values varying from 22.9 to 34.0 μM, and irpexoate D (**4**) showed weak cytotoxicity against the human cancer cell line SW480 with an IC_50_ value of 35.2 μM.

**Graphical Abstract:**

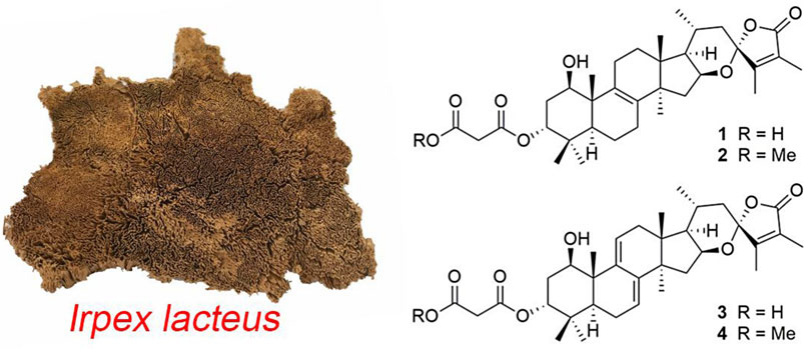

**Electronic supplementary material:**

The online version of this article (10.1007/s13659-018-0160-3) contains supplementary material, which is available to authorized users.

## Introduction

Natural products from higher fungi (mushroom) are regarded as a vital source of lead compounds in the field of drug research and development [1]. The fungus *Irpex lacteus* is widely used in China as a folk medicine [2]. Previously chemical investigation on this fungus major focused on secondary metabolites from culture broth [3, 4], while the study on the fruiting bodies remained untapped.

**Fig. 1:**
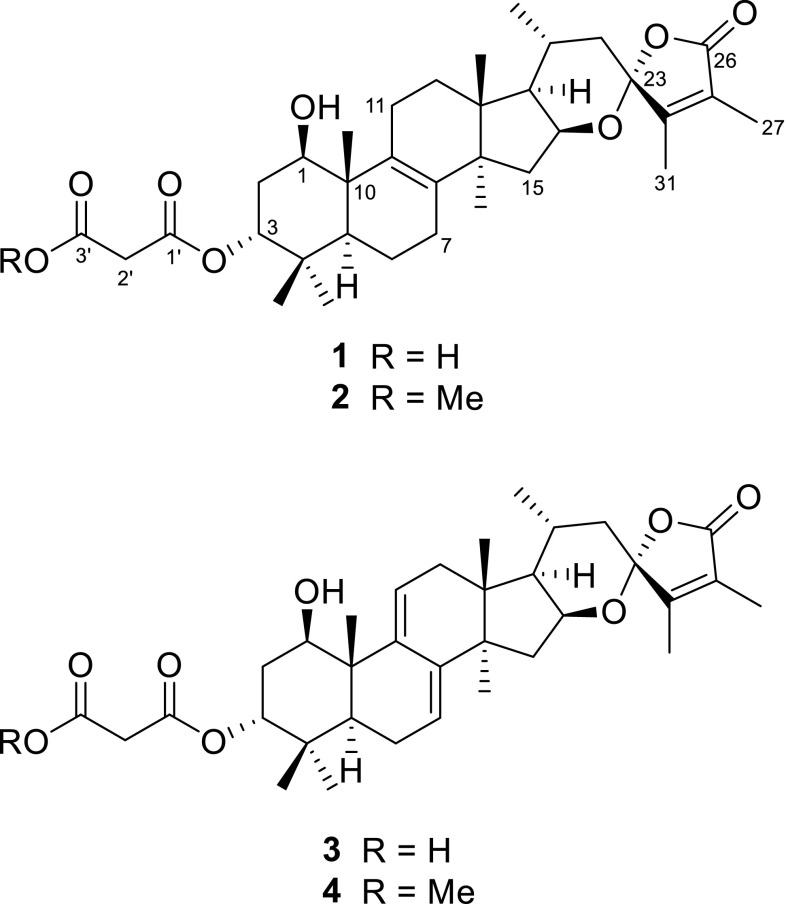
Chemical structures of compounds **1**–**4**

Triterpenoids are one of the largest groups of secondary metabolites from higher fungi. To the best of our knowledge, lanostane-type triterpenoids are by far the most diverse category of higher fungi-derived triterpenoids. This type of triterpenes always encounters in the genus *Ganoderma* and the fungus *Poria cocos* [1]. However, eburicane-type triterpenes, which also can be considered as 24-methyl lanostanes, have rarely been found from fungi. Triterpenoids with malonyl modification are not prevalent in fungal metabolites. From biosynthetic point, the malonyl moieties were introduced by the precursor malonyl-CoA, which was a key intermediate in the pathways of fatty acid biosynthesis and fatty acid elongation, and also a key signaling molecule in mammalian cells [5]. As our continuous efforts to search promising lead compounds from mushroom, four malonyl modified eburicanes, namely irpexoates A–D (**1**–**4**) (Fig. 1), were isolated from the fruiting bodies of the medicinal fungus *I. lacteus*. Herein, we report the isolation, structure elucidation, and cytotoxicity against five human cancer lines of the isolates irpexoates A–D (**1**–**4**).

## Results and Discussion

**Fig. 2:**
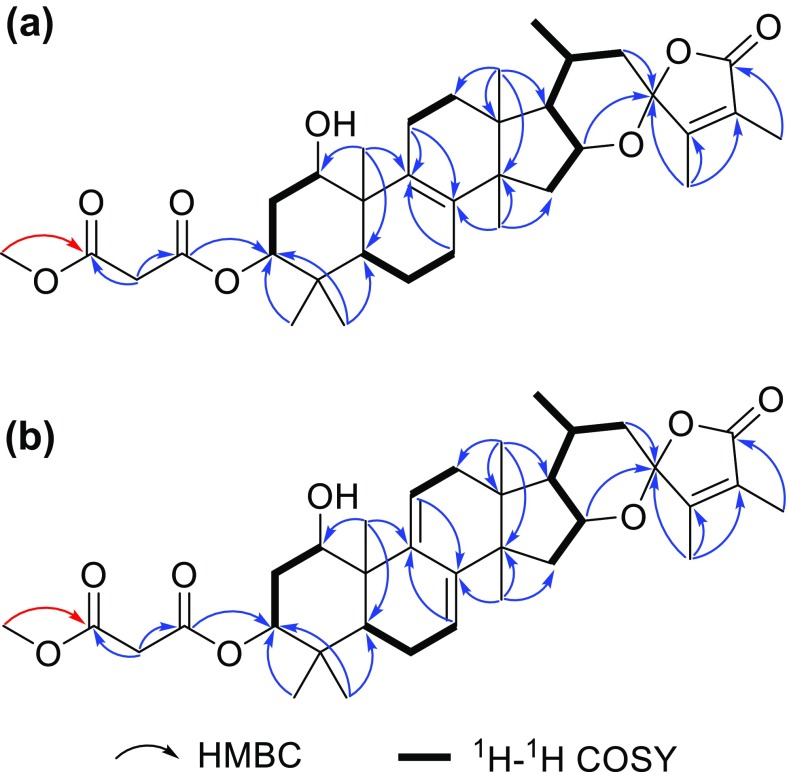
Characteristic mutual HMBC (blue arrow) and ^1^H–^1^H COSY correlations for **a** compounds **1** and **2** (HMBC in red arrow was exclusive for **2**) and **b** compounds **3** and **4** (HMBC in red arrow was exclusive for **4**)

Compound **1** was obtained as a white amorphous powder. It had a molecular formula of C_34_H_48_O_8_ as determined by (+)-HRESIMS protonated ion peak at *m/z* 585.3433 [M+H]^+^ (calcd for C_34_H_49_O_8_, 585.3422), corresponding to 11° of unsaturation. The IR spectrum showed absorption bands for hydroxy (3436 cm^−1^) and carbonyl (1764 cm^−1^) groups. The ^1^H NMR spectrum of **1** (Table 1) showed the presence of seven methyl singlets at *δ*_H_ 0.86 (CH_3_-18), 1.03 (CH_3_-19), 1.78 (CH_3_-27), 0.87 (CH_3_-28), 0.93 (CH_3_-29), 0.95 (CH_3_-30), and 1.94 (CH_3_-31), and one doublet methyl at *δ*_H_ 1.02 (*J* = 6.0 Hz, CH_3_-21). The ^13^C NMR and DEPT spectra presented 34 carbons ascribable to eight methyls, eight methylenes, six methines, and twelve quaternary carbons, including four sp^3^ ones, four olefinic ones, three carbonyls, and a ketal carbon (*δ*_C_ 107.7, C-23). All these spectroscopic features implied the presence of an eburicane skeleton in compound **1**. Exhaustive analysis of the 2D NMR spectra furnished the establishment of the structure of **1**. The HMBC correlations from H_3_-31 to C-23, C-24 (*δ*_C_ 157.8), and C-25 (*δ*_C_ 124.1), from H_3_-27 to C-24, C-25, and C-26 (*δ*_C_ 172.5), from H-16 (*δ*_H_ 4.51) to C-23 (Fig. 2) indicated the existence of a 26,23-lactone, and a 16,23-epoxy group, which constructing a spiro ring system with C-23 as the shared carbon. The HMBC correlations from H_3_-19 to C-1 (*δ*_C_ 71.3), C-9 (*δ*_C_ 135.1), from H_3_-28, H_3_-29 to C-3 (*δ*_C_ 81.0), and from H_3_-30 to C-8 (*δ*_C_ 134.8) (Fig. 2) revealed that C-1 and C-3 were oxygenated, and a double bond was assigned at the positions C-8–C-9. Apart from the signals assigned to the eburicane skeleton, the remained three carbon signals, i.e. two carbonyls at *δ*_C_ 167.8 (C-1′) and 169.7 (C-3′), and a methylene at *δ*_C_ 41.1 (C-2′) were due to a malonyl moiety attached to C-3, which was supported by the HMBC correlations from H-2′ (*δ*_H_ 3.46; 3.40) to C-1′, C-3′, and from H-3 (*δ*_H_ 4.83) to C-1′.

**Table 1:** ^1^H NMR spectroscopic data for compounds **1**–**4** (CDCl_3_)

No.	**1** ^a^	**2** ^a^	**3** ^a^	**4** ^b^
1	3.82, dd (9.0, 6.4)	3.80, dd (10.0, 6.3)	4.10, dd (10.0, 6.3)	4.09, dd (10.0, 6.3)
2	1.92, overlapped	1.92, m	1.98, overlapped	1.96, overlapped
3	4.83, br t (3.0)	4.81, t (3.0)	4.85, br t (3.0)	4.80, t (3.0)
5	1.46, dd (10.0, 6.0)	1.44, dd (10.0, 6.0)	1.51, dd (12.0, 4.0)	1.49, dd (12.0, 4.0)
6	1.63, overlapped	1.64, overlapped	2.15, overlapped 2.06, overlapped	2.14, dd (18.8, 14.0) 2.06, overlapped
7	2.09, m 2.04, m	2.10, overlapped 2.04, overlapped	5.49, d (6.5)	5.49, d (6.3)
11	2.43, m 2.21, br dd (19.0, 8.5)	2.44, overlapped 2.23, overlapped	6.30, d (6.0)	6.30, d (6.0)
12	1.84, overlapped 1.62, overlapped	1.85, overlapped 1.64, overlapped	2.24, d (17.5) 2.08, overlapped	2.24, d (17.7) 2.09, overlapped
15	1.88, dd (12.4, 8.0) 1.70, dd (12.4, 6.5)	1.88, dd (12.5, 7.8) 1.71, dd (12.5, 6.5)	2.06, overlapped 1.71, dd (12.5, 6.5)	2.06, overlapped 1.71, dd (12.5, 6.5)
16	4.51, ddd (8.0, 8.0, 6.5)	4.51, ddd (7.8, 7.8, 6.5)	4.59, ddd (7.8, 7.8, 6.5)	4.59, ddd (7.8, 7.8, 6.5)
17	1.59, overlapped	1.59, overlapped	1.67, overlapped	1.66, m
18	0.86, s	0.86, s	0.75, s	0.75, s
19	1.03, s	1.02, s	1.07, s	1.07, s
20	1.81, overlapped	1.81, overlapped	1.82, overlapped	1.81, overlapped
21	1.02, d (6.0)	1.03, d (6.0)	1.03, d (6.5)	1.02, d (6.5)
22	1.81, overlapped	1.81, overlapped	1.82, overlapped	1.82, overlapped
27	1.78, s	1.78, s	1.78, s	1.78, s
28	0.87, s	0.87, s	0.89, s	0.88, s
29	0.93, s	0.93, s	1.01, s	1.00, s
30	0.95, s	0.94, s	0.93, s	0.92, s
31	1.94, s	1.94, s	1.93, s	1.93, s
2′	3.46, d (16.5) 3.40, d (16.5)	3.43, d (16.9) 3.39, d (16.9)	3.46, d (17.0) 3.41, d (17.0)	3.46, d (17.0) 3.41, d (17.0)
4′		3.73, s		3.70, s

^a^Measured at 500 MHz

^b^Measured at 600 MHz

**Fig. 3:**
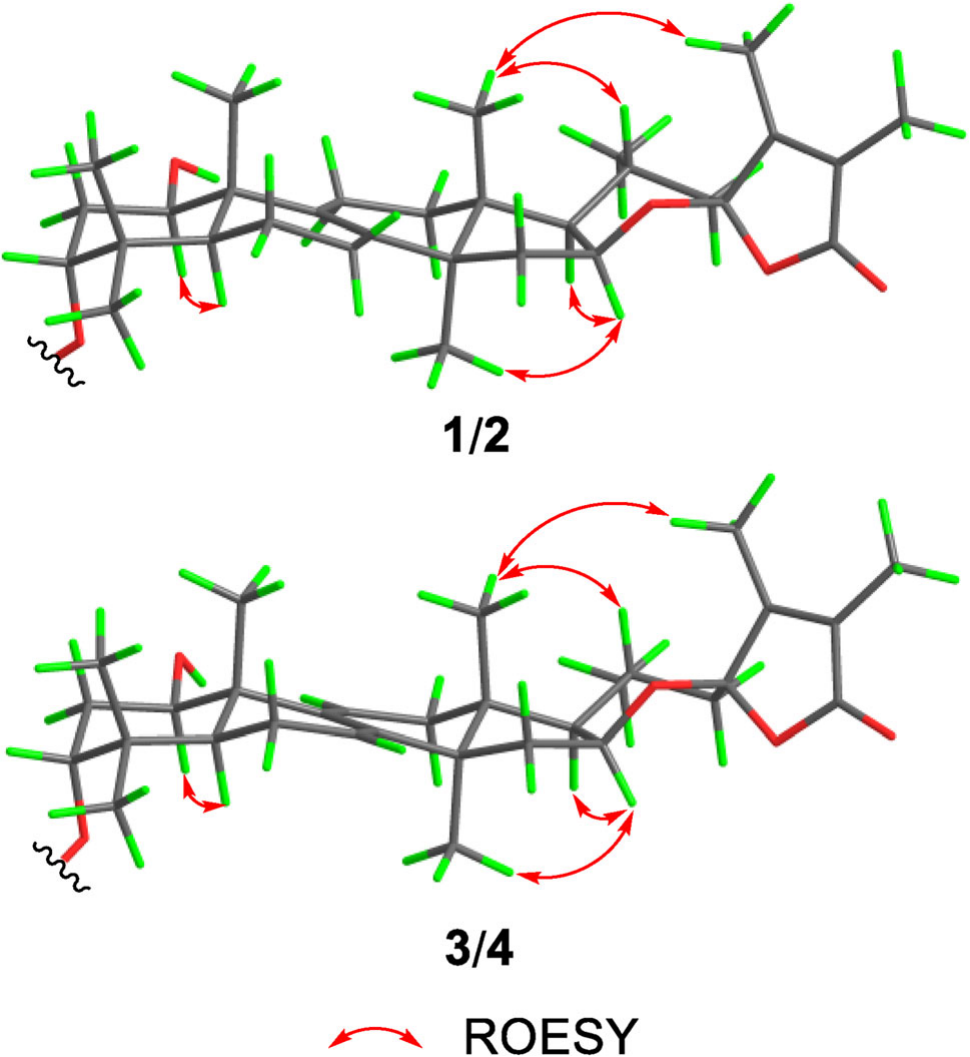
Key ROESY correlations for compounds **1**–**4**

The ROESY correlations between H-1/H-5, H_3_-30/H-16 demonstrated that 1-OH was *β* orientation while H-16 was *α* orientation. The key diagnostic ROESY correlation between H_3_-18 and H_3_-31 revealed that C-23 was R* configuration (Fig. 3). As for the configuration of C-3, the broad triplet of H-3 with a small coupling constant (3.0 Hz) indicated the *β* orientation for H-3, which can be seen as a consequence of the nearly same dihedral angle between H-3/H-2*α*, and H-3/H-2*β* when H-3 adopted *β* orientation (Fig. 3). Therefore, compound **1** was determined as shown in Fig. 1, and given the name irpexoate A.

The white amorphous powder compound **2**, named irpexoate B, possessed the molecular formula of C_35_H_50_O_8_ as determined by the (+)-HRESIMS sodium adduct ion peak at *m/z* 621.3399 [M+Na]^+^ (calcd for C_35_H_50_O_8_Na, 621.3398), indicating 11 indices of hydrogen deficiency. The ^1^H and ^13^C NMR spectra of **2** (Tables 1, 2) showed highly similarities to those of **1**, indicating that these two compounds possessed identical carbon scaffold and substitution patterns. Compared to **1**, the presence of an additional methoxy group at *δ*_C_ 52.4 (C-4′) in **2**, along with the HMBC correlation from H_3_-4′ (*δ*_H_ 3.72) to C-3′ (*δ*_C_ 167.1) (Fig. 2) revealed that the terminal carboxylic acid group was methyl-esterified in **2**. Other characteristic ROESY correlations suggested that the stereochemistry of chiral centers in **2** was identical with those of **1**. As describe above, compound **2** was determined as shown in Fig. 1.

**Table 2:** ^13^C NMR spectroscopic data for compounds **1**–**4** (CDCl_3_)

No.	**1** ^a^	**2** ^a^	**3** ^a^	**4** ^b^
1	71.3, CH	71.3, CH	71.6, CH	71.6, CH
2	33.4, CH_2_	33.5, CH_2_	33.5, CH_2_	33.7, CH_2_
3	81.0, CH	80.4, CH	81.0, CH	80.3, CH
4	36.5, C	36.6, C	36.4, C	36.5, C
5	44.4, CH	44.3, CH	43.9, CH	43.9, CH
6	17.9, CH_2_	17.8, CH_2_	23.1, CH_2_	23.1, CH_2_
7	26.0, CH_2_	26.1, CH_2_	120.2, CH	120.4, CH
8	134.8, C	134.8, C	141.8, C	141.8, C
9	135.1, C	135.2, C	144.2, C	144.3, C
10	43.0, C	43.1, C	43.4, C	43.5, C
11	23.9, CH_2_	23.9, CH_2_	119.8, CH	119.8, CH
12	31.6, CH_2_	31.6, CH_2_	37.6, CH_2_	37.6, CH_2_
13	43.4, C	43.4, C	42.8, C	42.8, C
14	47.4, C	47.4, C	47.9, C	48.0, C
15	39.5, CH_2_	39.5, CH_2_	40.0, CH_2_	40.1, CH_2_
16	75.5, CH	75.4, CH	75.2, CH	75.2, CH
17	53.0, CH	53.0, CH	53.5, CH	53.5, CH
18	18.4, CH_3_	18.3, CH_3_	18.4, CH_3_	18.4, CH_3_
19	13.8, CH_3_	13.7, CH_3_	15.2, CH_3_	15.2, CH_3_
20	26.3, CH	26.3, CH	26.1, CH	26.1, CH
21	20.8, CH_3_	20.8, CH_3_	20.6, CH_3_	20.6, CH_3_
22	37.5, CH_2_	37.5, CH_2_	37.4, CH_2_	37.5, CH_2_
23	107.7, C	107.6, C	107.6, C	107.5, C
24	157.8, C	157.8, C	157.7, C	157.7, C
25	124.1, C	124.1, C	124.1, C	124.1, C
26	172.5, C	172.5, C	172.5, C	172.3, C
27	8.3, CH_3_	8.3, CH_3_	8.3, CH_3_	8.3, CH_3_
28	27.2, CH_3_	27.2, CH_3_	27.5, CH_3_	27.4, CH_3_
29	21.5, CH_3_	21.5, CH_3_	22.2, CH_3_	22.2, CH_3_
30	25.2, CH_3_	25.2, CH_3_	25.9, CH_3_	25.8.CH_3_
31	10.9, CH_3_	10.9, CH_3_	10.8, CH_3_	10.9, CH_3_
1′	167.8, C	165.9, C	167.7, C	165.9, C
2′	41.1, CH_2_	41.7, CH_2_	40.4, CH_2_	41.6, CH_2_
3′	169.7, C	167.1, C	168.1, C	166.8, C
4′		52.4, CH_3_		52.4, CH_3_

^a^Measured at 125 MHz

^b^Measured at 150 MHz

Compound **3** gave a molecular formula of C_34_H_46_O_8_ according to the ^13^C NMR data and the (+)-HRESIMS sodium adduct ion peak at *m/z* 605.3058 [M+Na]^+^ (calcd for C_34_H_46_O_8_Na, 605.3085). A comparison of the ^1^H and ^13^C NMR data of **3** to those of **1** (Tables 1 , 2) revealed that **3** was an analogue of **1**. Carefully analysis of the HMBC and ^1^H–^1^H COSY spectra of **3** revealed the existence of a conjugated diene locating at C-7–C-8–C-9–C-11, which was ascertainable by the HMBC correlations from the olefinic protons H-7 (*δ*_H_ 5.49) and H-11 (*δ*_H_ 6.30) to C-8 (*δ*_C_ 141.8), C-9 (*δ*_C_ 144.2) as well as the ^1^H–^1^H COSY cross peaks between H-6/H-7, H-11/H-12 (Fig. 2). The stereochemistry of **3** was assigned by a ROESY experiment and coupling constants analysis. The diagnostic ROESY correlations between H-1/H-5, H_3_-18/H-20, H_3_-18/H_3_-31, H_3_-30/H-16/H-17 indicated that both H-1 and H-16 were *α* orientation, and the spiro carbon C-23 was R* configuration (Fig. 3). The triplet peak of H-3 (*δ*_H_ 4.85) with small coupling constant (3.0 Hz) suggested the malonate moiety at the axial position. Therefore, compound **3** was established as shown in Fig. 1 and given the trivial name irpexoate C.

A molecular formula of C_35_H_48_O_8_ was assigned to compound **4** as determined by the (+)-HRESIMS sodium adduct ion peak at *m/z* 619.3250 [M+Na]^+^ (calcd for C_35_H_48_O_8_Na, 619.3241). Comparison of the 1D NMR data of **4** (Tables 1, 2) to those of **3** showed that they were closely related analogues featuring identical carbon frameworks. 2D NMR spectra analysis of **4** indicated that the main distinction was attributable to the substituent group at C-3. The HMBC correlations from H-3 (*δ*_H_ 4.80) to C-1′ (*δ*_C_ 165.9), from H-2′ (*δ*_H_ 3.41, 3.46) to C-1′ and C-3′ (*δ*_C_ 166.8), and CH_3_-4′ (*δ*_H_ 3.70) to C-3′ allowed a methyl malonate moiety linked at C-3. The relative configuration of **4** was consistent with that of **3** as established by the ROESY spectrum. The structure of **4** was consequently assigned as shown in Fig. 1, and named as irpexoate D.

Irpexoates A–D (**1**–**4**) were evaluated for the cytotoxicity against five human cancer cell lines (the human myeloid leukemia cell line HL-60, the human hepatocellular carcinoma cell line SMMC-7721, the lung cancer cell line A549, the breast cancer cell line MCF-7, and the human colon cancer cell line SW-480). The results showed that compound **2** exhibited weak cytotoxicity against the four human cancer cell lines (A-549, SMMC-7721, MCF-7, SW480) with IC_50_ values varying from 22.9 to 34.0 μM, while compound **4** only showed weak cytotoxicity against the human colon cancer cell line SW480 with an IC_50_ value of 11.2 ± 0.25 μM (Table 3). However, compounds **1** and **3** were devoid of cytotoxicity. Thus, the conclusion could be drawn that the absence of methoxy group in the malonyl moiety attenuated the cytotoxicity.

**Table 3:** Cytotoxicity of compounds **2** and **4** against five human cancer cell lines (IC_50_ ± SD in μM)

Compound	HL-60	A-549	SMMC-7721	MCF-7	SW480
**2**	> 40	22.9 ± 0.92	30.1 ± 0.43	24.2 ± 1.11	34.0 ± 1.43
**4**	> 40	> 40	> 40	> 40	35.2 ± 1.15
Cisplatin		14.1 ± 0.11	11.9 ± 0.49	14.1 ± 1.00	11.2 ± 0.25

## Experimental

### General Experimental Procedures

Optical rotations were obtained on a JASCO P-1020 digital polarimeter (Horiba, Kyoto, Japan). UV spectra were recorded on a Shimadzu UV-2401PC UV–visible recording spectrophotometer (Shimadzu, Kyoto, Japan). A Tenor 27 spectrophotometer (Bruker Optics GmbH, Ettlingen, Germany) was used for scanning IR spectroscopy using KBr pellets. 1D and 2D NMR spectra were obtained on Bruker DRX 500 MHz and Avance III 600 MHz spectrometers (Bruker Corporation, Karlsruhe, Germany). HRESIMS were recorded on an Agilent 6200 Q-TOF MS system (Agilent Technologies, Santa Clara, CA, USA). Sephadex LH-20 (Amersham Biosciences, Uppsala, Sweden) and silica gel (Qingdao Haiyang Chemical Co., Ltd, Qingdao, China) were used for column chromatography (CC). Medium pressure liquid chromatography (MPLC) was performed on a Büchi Sepacore System equipped with pump manager C-615, pump modules C-605 and fraction collector C-660 (Büchi Labortechnik AG, Flawil, Switzerland), and columns packed with Chromatorex C-18 (dimensions 450 mm × i.d. 14 mm, particle size: 40–75 μm, Fuji Silysia Chemical Ltd., Kasugai, Japan). Preparative high performance liquid chromatography (prep-HPLC) were performed on an Agilent 1260 liquid chromatography system equipped with a Zorbax SB-C18 column (particle size 5 μm, dimension 150 mm × i.d. 9.4 mm, flow rate 7 ml min^−1^, respectively) and a DAD detector (Agilent Technologies).

### Fungal Material

The fungus *Irpex lacteus* was collected from the Wangtianshu Scenic Area, Xishuangbanna, Yunnan Province in July 2014, and authenticated by Prof. Yu-Cheng Dai (Beijing Forestry University), who is a mushroom specialist. A voucher specimen of *I. lacteus* was deposited at the Mushroom Bioactive Natural Products Research Group in Kunming Institute of Botany (No. HFG 201407).

### Extraction and Isolation

The dry fruiting bodies of *I. lacteus* (1.47 kg) was pulverized and macerated five times with 95% EtOH at room temperature. The extract was evaporated under reduced pressure and partitioned between EtOAc and water for three times to give an EtOAc layer (53 g). The crude extract was eluted on MPLC with a stepwise gradient of MeOH/H_2_O (20–100%) to afford eight fractions (A–H).

Fraction E was applied to silica gel column chromatography eluting with petroleum ether/acetone (5:1–2:1) to give ten subfractions (E1–E10). Subfraction E5 was purified by prep-HPLC (MeCN/H_2_O: 45–65%, 7 mL min^−1^, 20 min) to yield compound **4** (1.8 mg, t_*R*_ = 13.8 min). Fraction F was subjected to normal silica gel CC (CHCl_3_/MeOH, 40:1–1:1) to furnish seven subfractions (F1–F7). Subfraction F6 was purified on prep-HPLC (MeCN/H_2_O: 65%, isocratic, 18 mL min^−1^, 20 min) to yield compound **1** (26.6 mg, t_*R*_ = 9.5 min). Compound **2** (17.0 mg, t_*R*_ = 12.9 min) was purified from subfraction F1 by prep-HPLC (MeCN/H_2_O: 55–75%, 7 mL min^−1^, 20 min). Subfraction F4 was fractionated into five subfractions (F4a–F4e) by Sephadex LH-20, eluting with CHCl_3_–MeOH (1:1). Subfraction F4e was separated using silica gel CC (CHCl_3_/MeOH, 100:1 –10:1) to afford eight subfractions (F4e1–F4e8). Subfraction F4e5 was fractionated by Sephadex LH-20 (acetone) to give six subfractions (F4e5a–F4e5f). Subfraction F4e5c was further purified using prep-HPLC with a solvent gradient of 45–65% MeCN in H_2_O over 20 min, flow rate 7 ml·min^−1^, to yield compound **3** (3.0 mg, t_*R*_ = 12.9 min).

### Spectroscopic Data of Compounds

#### Irpexoate A (**1**)

Amorphous white powder; $[\alpha ]_{\text{D}}^{24}$ −24.6 (*c* 0.15, MeOH); UV (MeOH) λ_max_ (log *ε*) 206.4 (4.23); IR (KBr) *ν*_max_ 3436, 2960, 2933, 2878, 1764, 1612, 1381, 1314, 1158, 1036, 958 cm^−1^; ^1^H NMR (500 MHz, CDCl_3_) and ^13^C NMR (125 MHz, CDCl_3_) data, see Tables 1 and 2; HRESIMS *m/z* 585.3433 [M+H]^+^ (calcd for C_34_H_49_O_8_, 585.3422).

#### Irpexoate B (**2**)

Amorphous white powder; $[\alpha ]_{\text{D}}^{24}$ −23.5 (*c* 0.15, MeOH); IR (KBr) *ν*_max_ 3439, 2959, 2878, 1753, 1631, 1439, 1313, 1033, 957 cm^−1^; ^1^H NMR (500 MHz, CDCl_3_) and ^13^C NMR (125 MHz, CDCl_3_) data, see Tables 1 and 2; HRESIMS *m/z* 621.3399 [M+Na]^+^ (calcd for C_35_H_50_O_8_Na, 621.3398).

#### Irpexoate C (**3**)

Amorphous white powder; $[\alpha ]_{\text{D}}^{24}$ −4.0 (*c* 0.15, MeOH); UV (MeOH) λ_max_ (log *ε*) 209.0 (4.17), 232.6 (4.05), 242.0 (4.06) and 250.0 (3.93); IR (KBr) *ν*_max_ 3438, 2963, 2932, 2883, 1765, 1633, 1315, 1156, 1038, 958 cm^−1^; ^1^H NMR (500 MHz, CDCl_3_) and ^13^C NMR (125 MHz, CDCl_3_) data, see Tables 1 and 2; HRESIMS *m/z* 605.3058 [M+Na]^+^ (calcd for C_34_H_46_O_8_Na, 605.3085).

#### Irpexoate D (**4**)

Amorphous white powder; $[\alpha ]_{\text{D}}^{24}$ −4.8 (*c* 0.09, MeOH); IR (KBr) *ν*_max_ 3444, 2961, 2928, 2884, 1752, 1636, 1444, 1314, 1153, 1036, 958 cm^−1^; ^1^H NMR (600 MHz, CDCl_3_) and ^13^C NMR (150 MHz, CDCl_3_) data, see Tables 1 and 2; HRESIMS *m/z* 619.3250 [M+Na]^+^ (calcd for C_35_H_48_O_8_Na, 619.3241).

### Cytotoxicity Assays

Human myeloid leukemia HL-60 cells, lung cancer A-549 cells, hepatocellular carcinoma SMMC-7721 cells, breast cancer MCF-7 cells, and colon cancer SW480 cell lines were used in the cytotoxic assay. All the cells were cultured in DMEM or RPMI-1640 medium (Hyclone, USA), supplemented with 10% fetal bovine serum (Hyclone, USA) in 5% CO_2_ at 37 °C. The cytotoxicity assay was performed according to the MTS (3-(4,5-dimethylthiazol-2-yl)-5(3-carboxymethoxyphenyl)-2-(4-sulfopheny)-2*H*-tetrazolium) method in 96-well microplates. Briefly, 100 μL of adherent cells was seeded into each well of 96-well cell culture plates and allowed to adhere for 12 h before drug addition, while suspended cells were seeded just before drug addition to an initial density of 1 × 105 cells ml^−1^. Each tumor cell line was exposed to the tested compounds dissolved in DMSO at various concentrations in triplicate for 48 h, with cisplatin (Sigma, USA) as a positive control. After compound treatment, cell viability was detected and a cell growth curve was graphed. IC_50_ values were calculated by Reed and Muench’s method [6].

## Electronic supplementary material

Below is the link to the electronic supplementary material.

Supplementary material 1 (DOCX 11171 kb)
