# Supplementary material for: Irpexoates A–D, Four Triterpenoids with Malonyl Modifications from the Fruiting Bodies of the Medicinal Fungus Irpex lacteus
Source: Nat Prod Bioprospect. 2018 Mar 28;8(3):171–6. doi: 10.1007/s13659-018-0160-3 (PMC5971031; doi:10.1007/s13659-018-0160-3)

**Supplementary material for**

**Irpexoates A–D, Four Triterpenoids with Malonyl Modifications from the Fruiting Bodies of the Medicinal Fungus *Irpex lacteus***

Yang Tang,^a,b,c^ Zhen-Zhu Zhao,^b^ Zheng-Hui Li,^b^ Tao Feng,^b^ He-Ping Chen,^b,^* Ji-Kai Liu^b,^*

*^a^State Key Laboratory of Phytochemistry and Plant Resources in West China, Kunming Institute of Botany, Chinese Academy of Sciences, Kunming 650201, People’s Republic of China*

*^b^School of Pharmaceutical Sciences, South-Central University for Nationalities, Wuhan 430074, People’s Republic of China*

*^c^University of Chinese Academy of Sciences, Beijing 100049, People’s Republic of China*

**Corresponding authors**

*E-mail: [chenhp@mail.scuec.edu.cn](mailto:chenhp@mail.scuec.edu.cn) (H.-P. Chen)

*E-mail: [jkliu@mail.kib.ac.cn](mailto:jkliu@mail.kib.ac.cn) (J.-K. Liu)

**Contents**

[Figure 1S. ^1^H NMR spectrum of **1** (500 MHz, CDCl_3_). 2](#_Toc509823969)

[Figure 2S. ^13^C NMR and DEPT spectra of **1** (125 MHz, CDCl_3_). 3](#_Toc509823970)

[Figure 3S. HSQC spectrum of **1**. 4](#_Toc509823971)

[Figure 4S. ^1^H-^1^H COSY spectrum of **1**. 5](#_Toc509823972)

[Figure 5S. HMBC spectrum of **1**. 6](#_Toc509823973)

[Figure 6S. ROESY spectrum of **1**. 7](#_Toc509823974)

[Figure 7S. (+)-HRESIMS report of **1**. 8](#_Toc509823975)

[Figure 8S. UV spectrum of **1**. 9](#_Toc509823976)

[Figure 9S. IR spectrum of **1**. 10](#_Toc509823977)

[Figure 10S. ^1^H NMR spectrum of **2** (500 MHz, CDCl_3_). 11](#_Toc509823978)

[Figure 11S. ^13^C NMR and DEPT spectra of **2** (125 MHz, CDCl_3_). 12](#_Toc509823979)

[Figure 12S. HSQC spectrum of **2**. 13](#_Toc509823980)

[Figure 13S. ^1^H-^1^H COSY spectrum of **2**. 14](#_Toc509823981)

[Figure 14S. HMBC spectrum of **2**. 15](#_Toc509823982)

[Figure 15S. ROESY spectrum of **2**. 16](#_Toc509823983)

[Figure 16S. (+)-HRESIMS report of **2**. 17](#_Toc509823984)

[Figure 17S. IR spectrum of **2**. 18](#_Toc509823985)

[Figure 18S. ^1^H NMR spectrum of **3** (500 MHz, CDCl_3_). 19](#_Toc509823986)

[Figure 19S. ^13^C NMR and DEPT spectra of **3** (125 MHz, CDCl_3_). 20](#_Toc509823987)

[Figure 20S. HSQC spectrum of **3**. 21](#_Toc509823988)

[Figure 21S. ^1^H-^1^H COSY spectrum of **3**. 22](#_Toc509823989)

[Figure 22S. HMBC spectrum of **3**. 23](#_Toc509823990)

[Figure 23S. ROESY spectrum of **3**. 24](#_Toc509823991)

[Figure 24S. (+)-HRESIMS report of **3**. 25](#_Toc509823992)

[Figure 25S. UV spectrum of **3**. 26](#_Toc509823993)

[Figure 26S. IR spectrum of **3**. 27](#_Toc509823994)

[Figure 27S. ^1^H NMR spectrum of **4** (600 MHz, CDCl_3_). 28](#_Toc509823995)

[Figure 28S. ^13^C NMR and DEPT spectra of **4** (150 MHz, CDCl_3_). 29](#_Toc509823996)

[Figure 29S. HSQC spectrum of **4**. 30](#_Toc509823997)

[Figure 30S. ^1^H-^1^H COSY spectrum of **4**. 31](#_Toc509823998)

[Figure 31S. HMBC spectrum of **4**. 32](#_Toc509823999)

[Figure 32S. ROESY spectrum of **4**. 33](#_Toc509824000)

[Figure 33S. (+)-HRESIMS report of **4**. 34](#_Toc509824001)

[Figure 34S. IR spectrum of **4**. 35](#_Toc509824002)

# Figure 1S. ^1^H NMR spectrum of **1** (500 MHz, CDCl_3_).


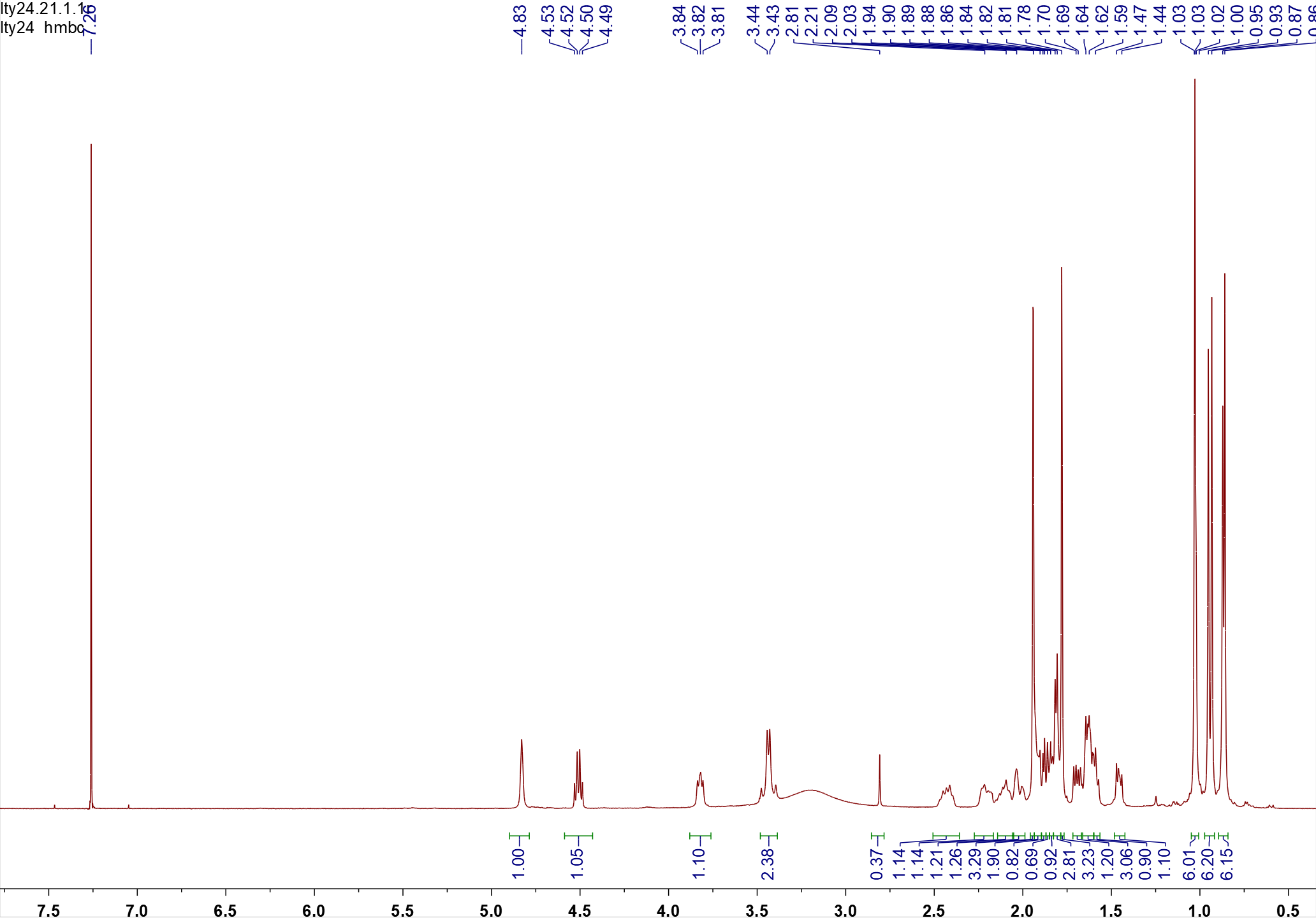


# Figure 2S. ^13^C NMR and DEPT spectra of **1** (125 MHz, CDCl_3_).


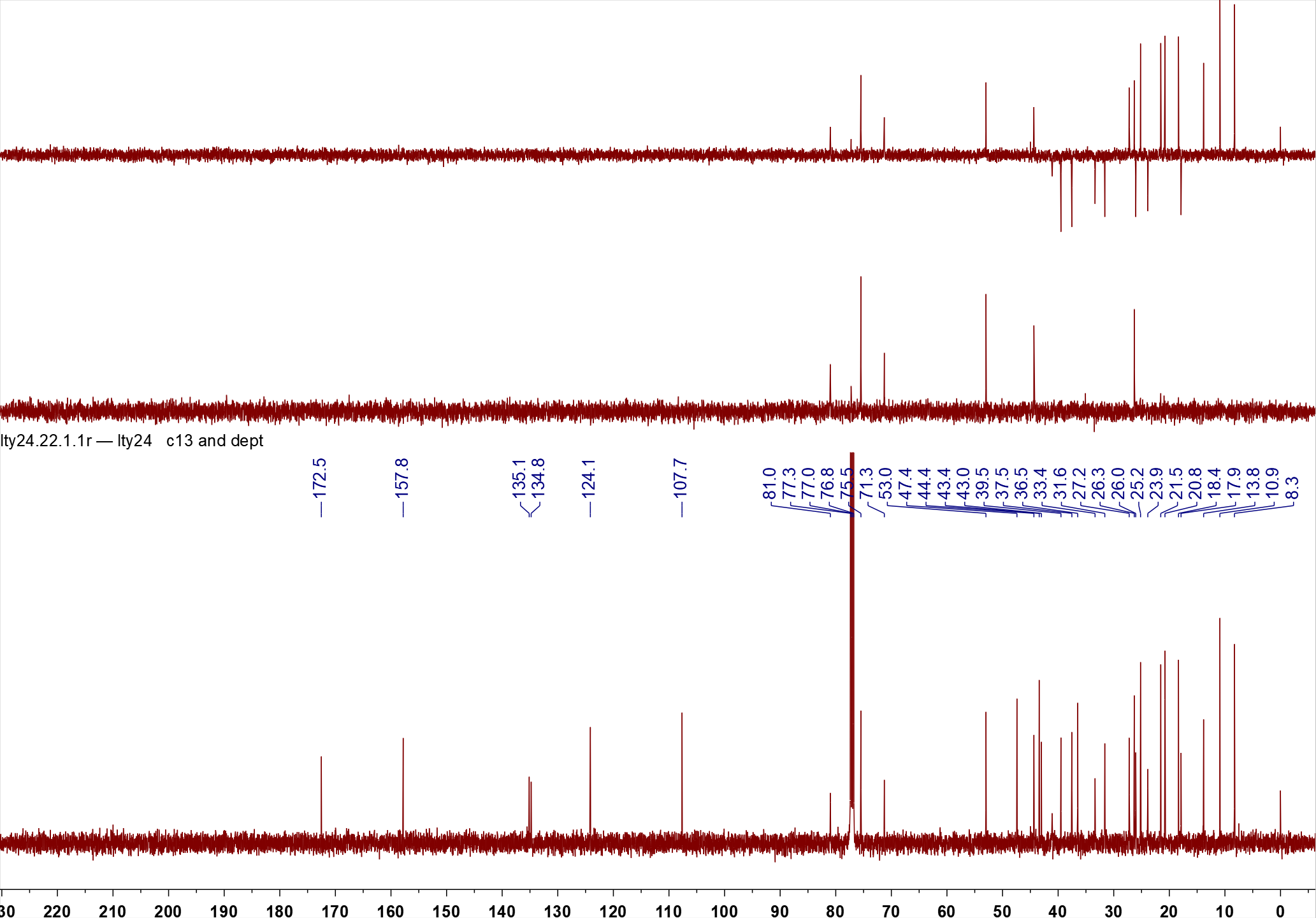


# Figure 3S. HSQC spectrum of **1**.


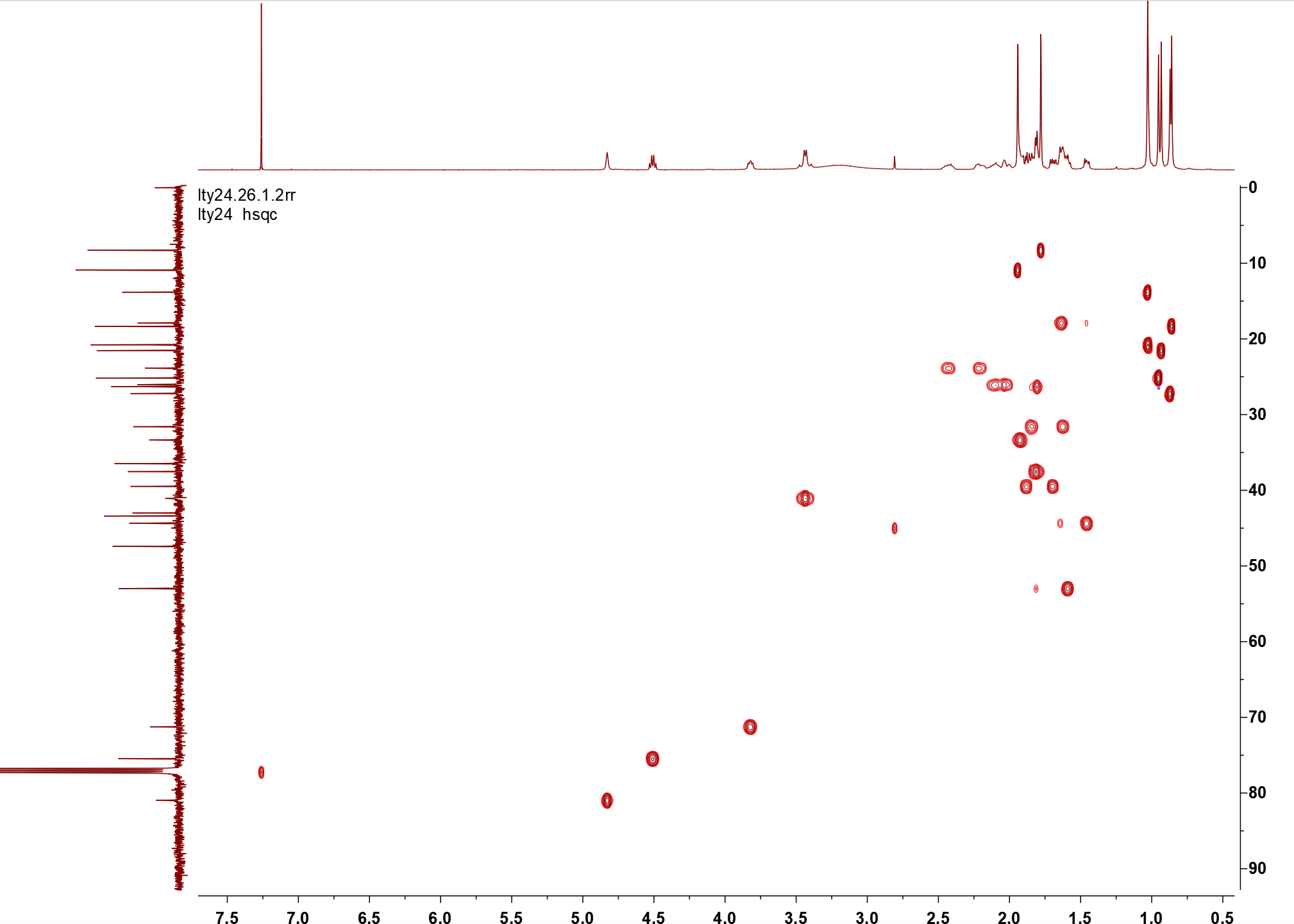


# Figure 4S. ^1^H-^1^H COSY spectrum of **1**.


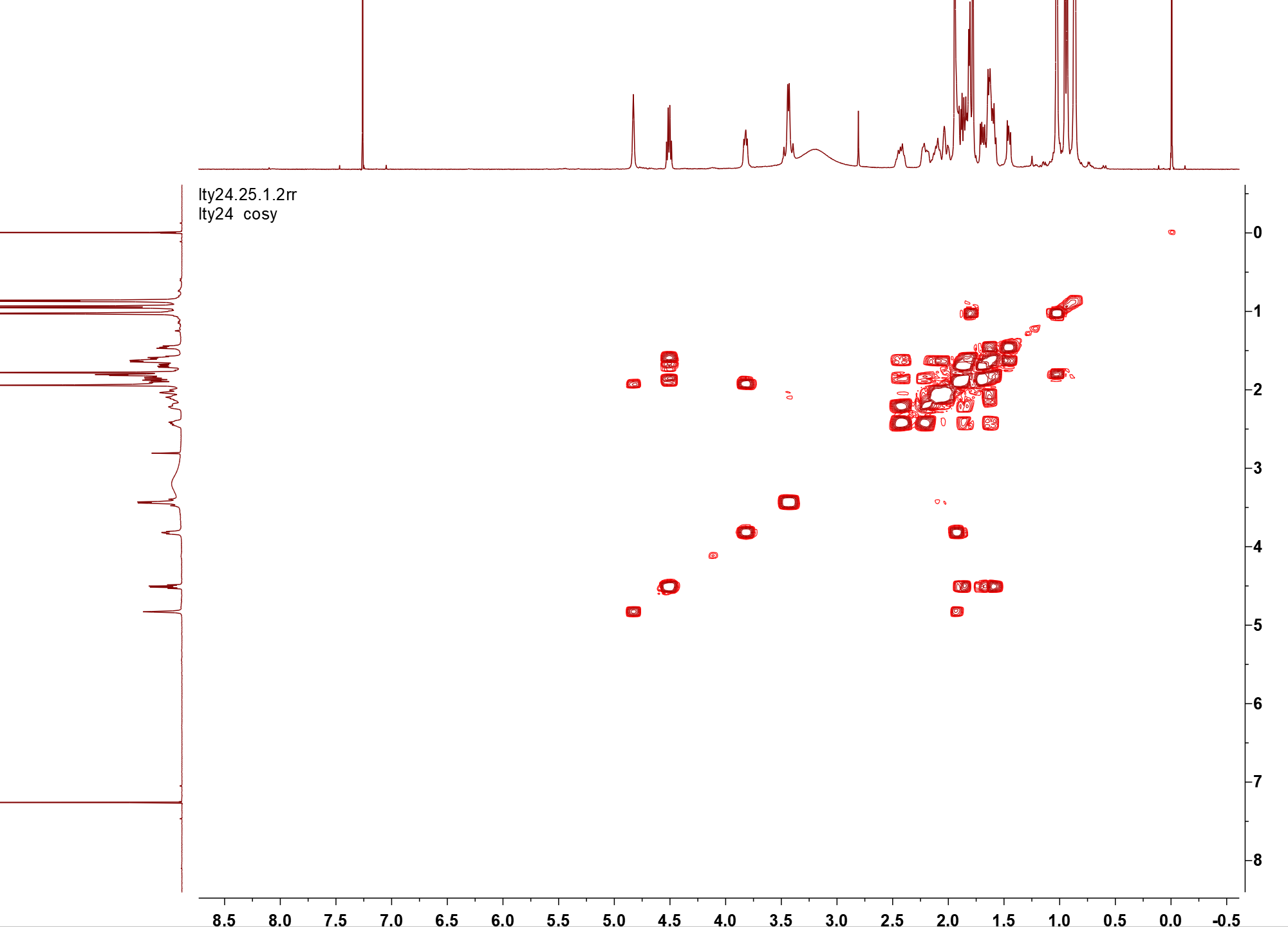


# Figure 5S. HMBC spectrum of **1**.


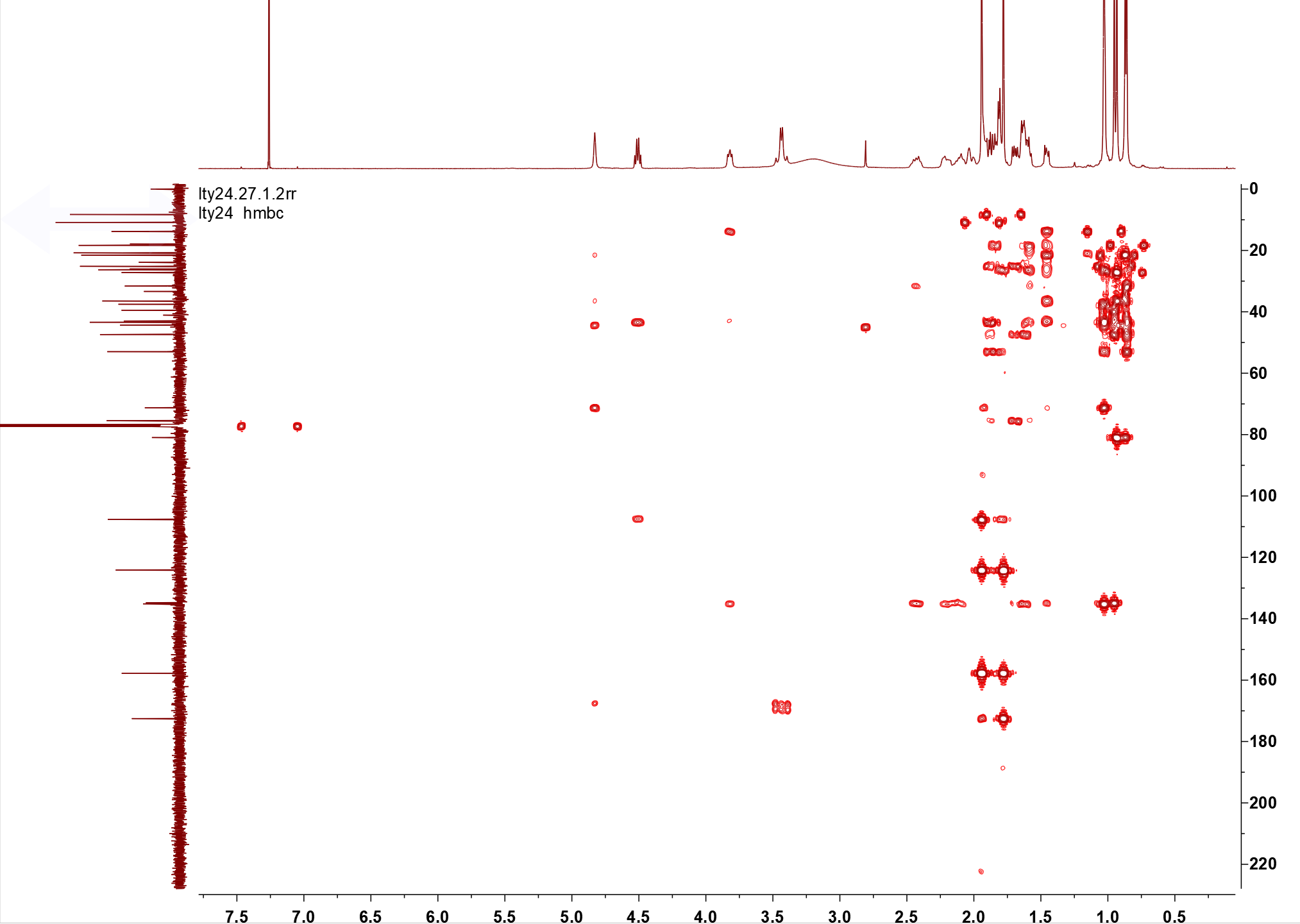


# Figure 6S. ROESY spectrum of **1**.


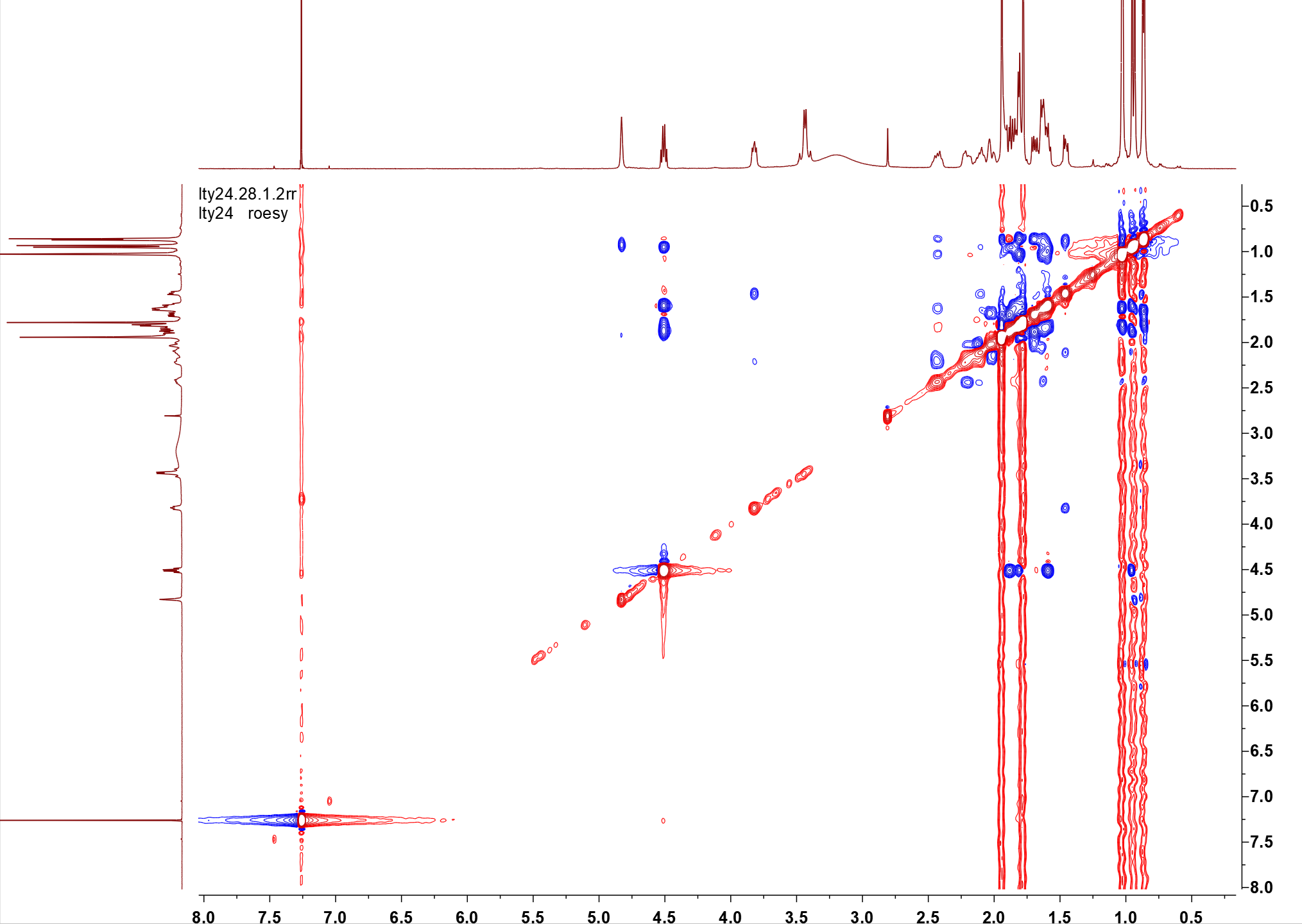


# Figure 7S. (+)-HRESIMS report of **1**.


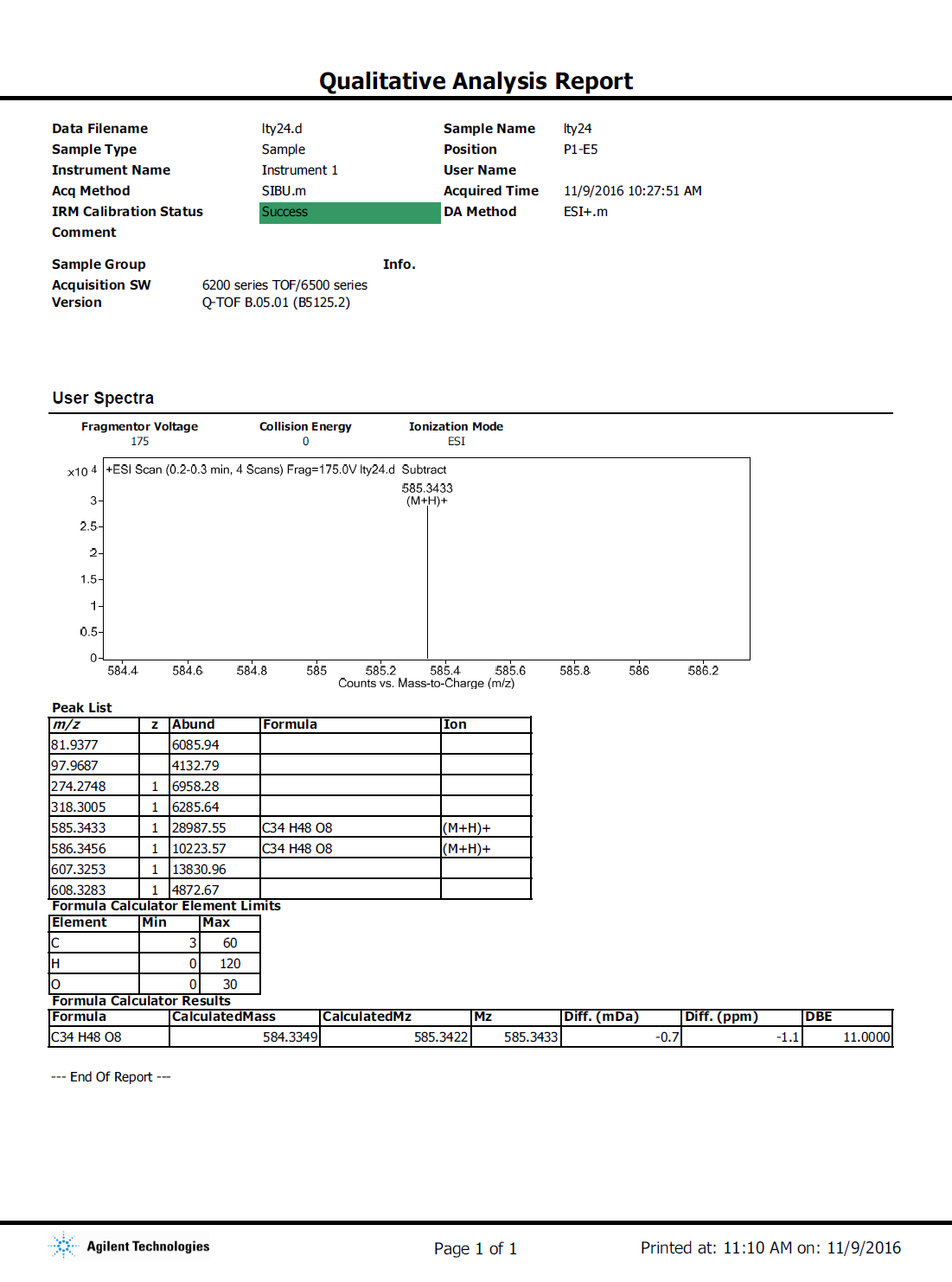


# Figure 8S. UV spectrum of **1**.


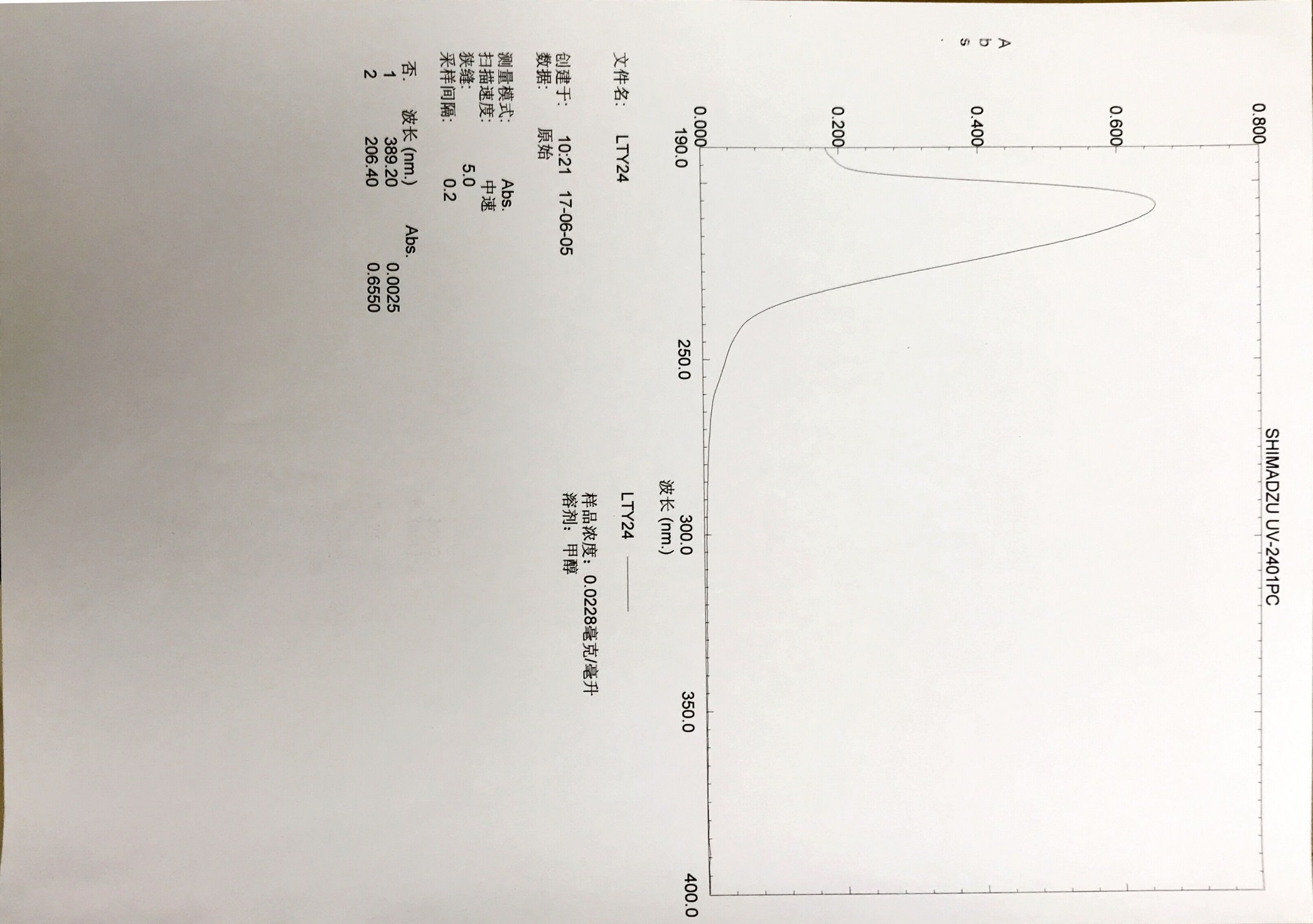


# Figure 9S. IR spectrum of **1**.


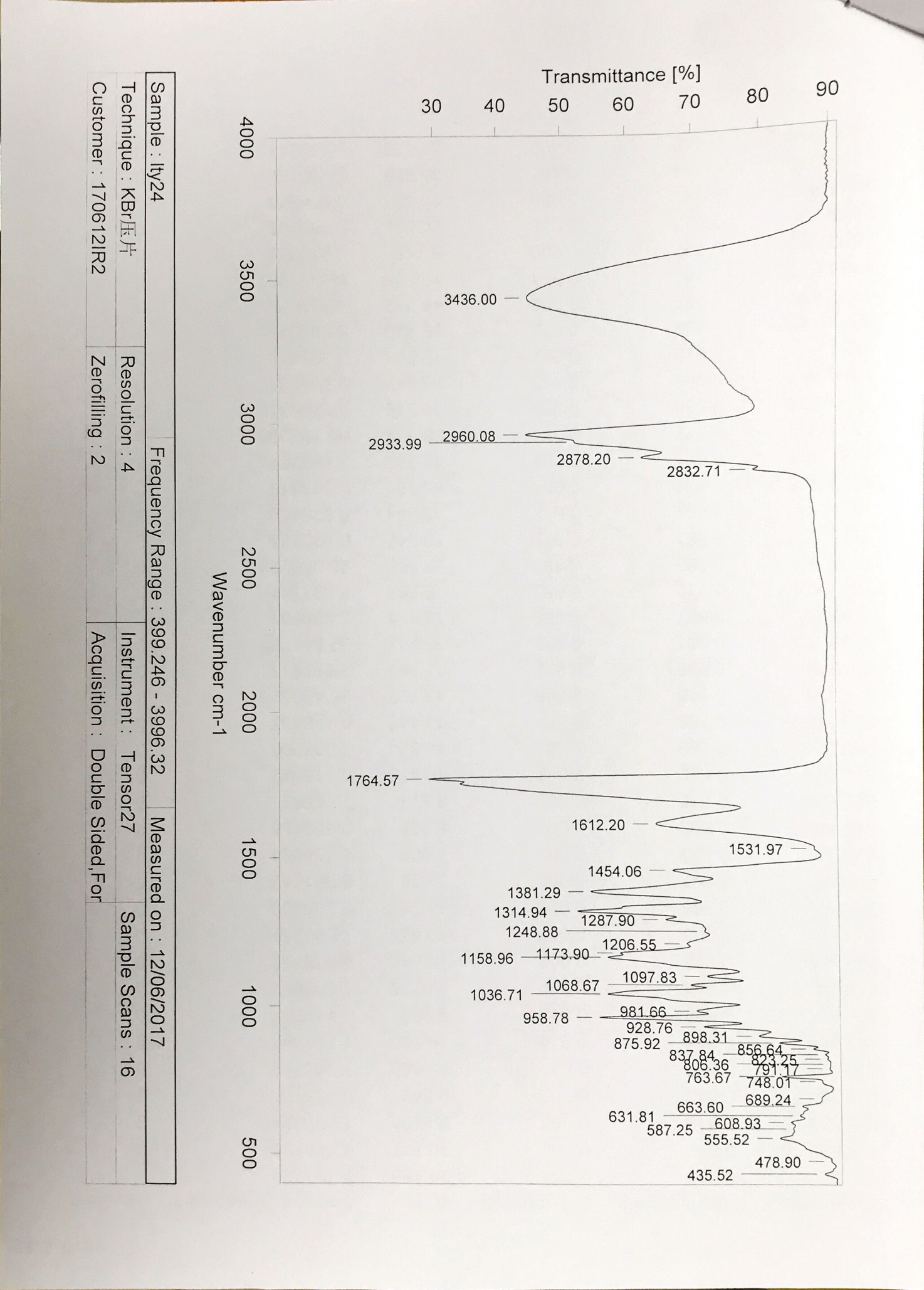


# Figure 10S. ^1^H NMR spectrum of **2** (500 MHz, CDCl_3_).


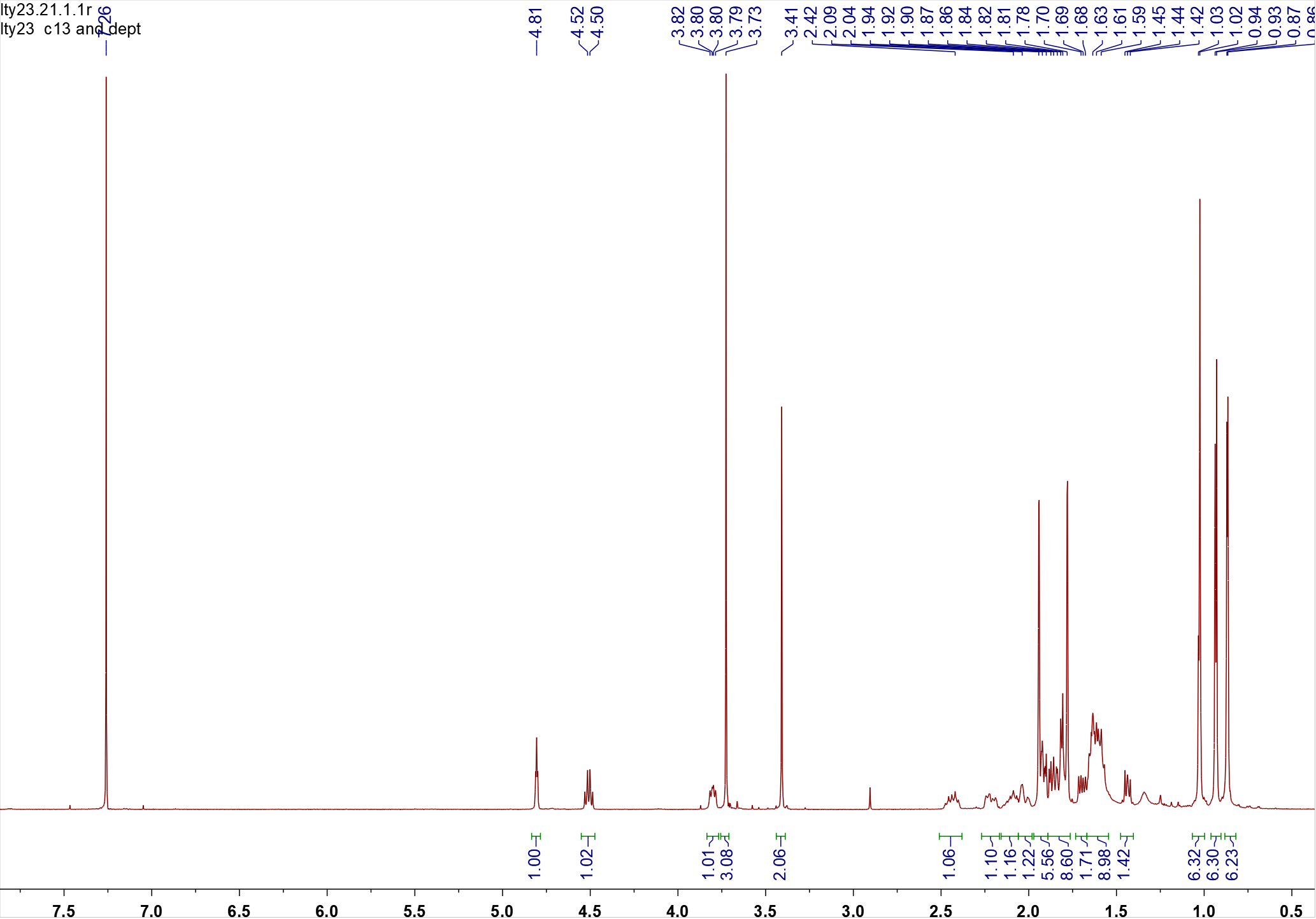


# Figure 11S. ^13^C NMR and DEPT spectra of **2** (125 MHz, CDCl_3_).


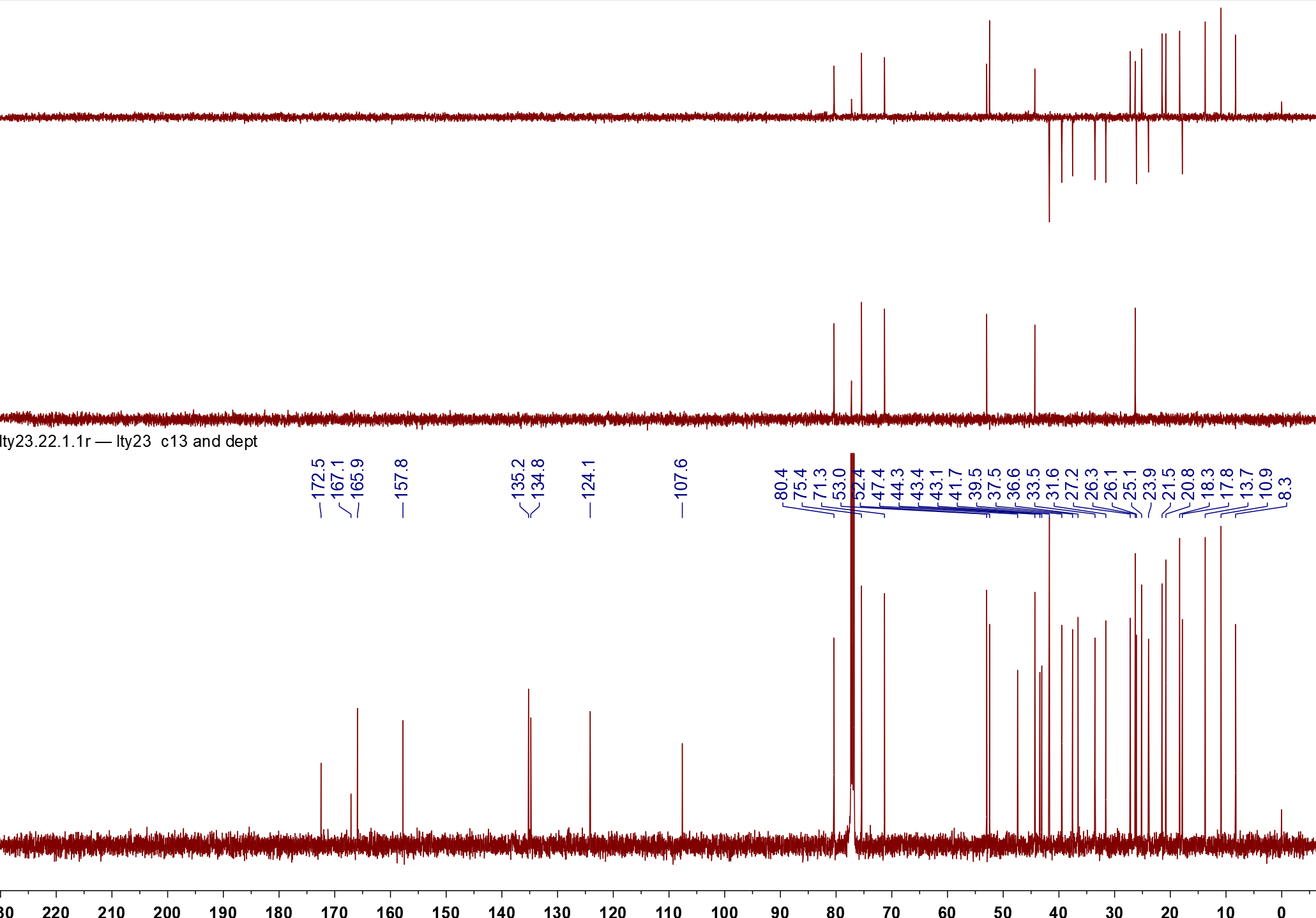


# Figure 12S. HSQC spectrum of **2**.


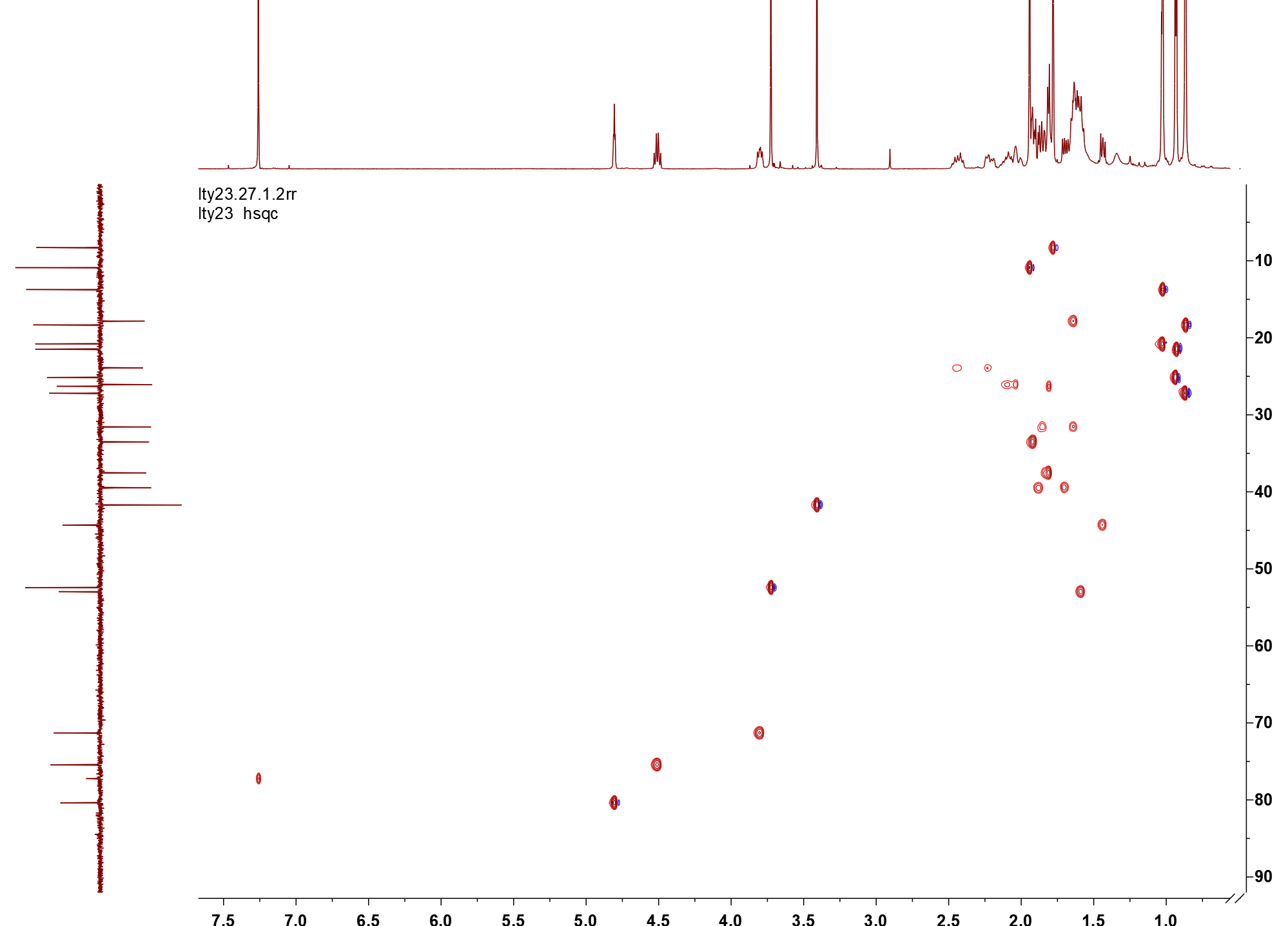


# Figure 13S. ^1^H-^1^H COSY spectrum of **2**.


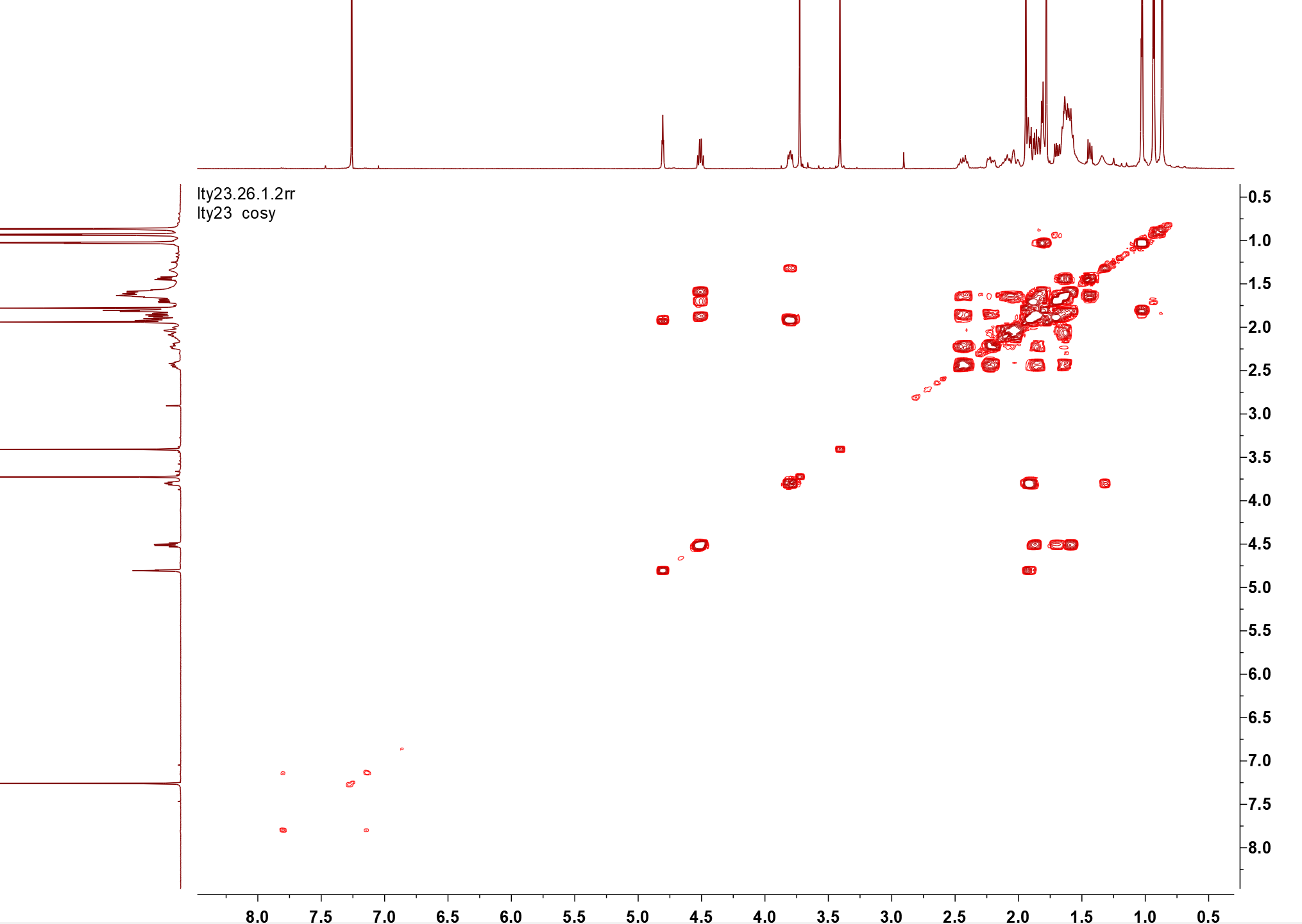


# Figure 14S. HMBC spectrum of **2**.


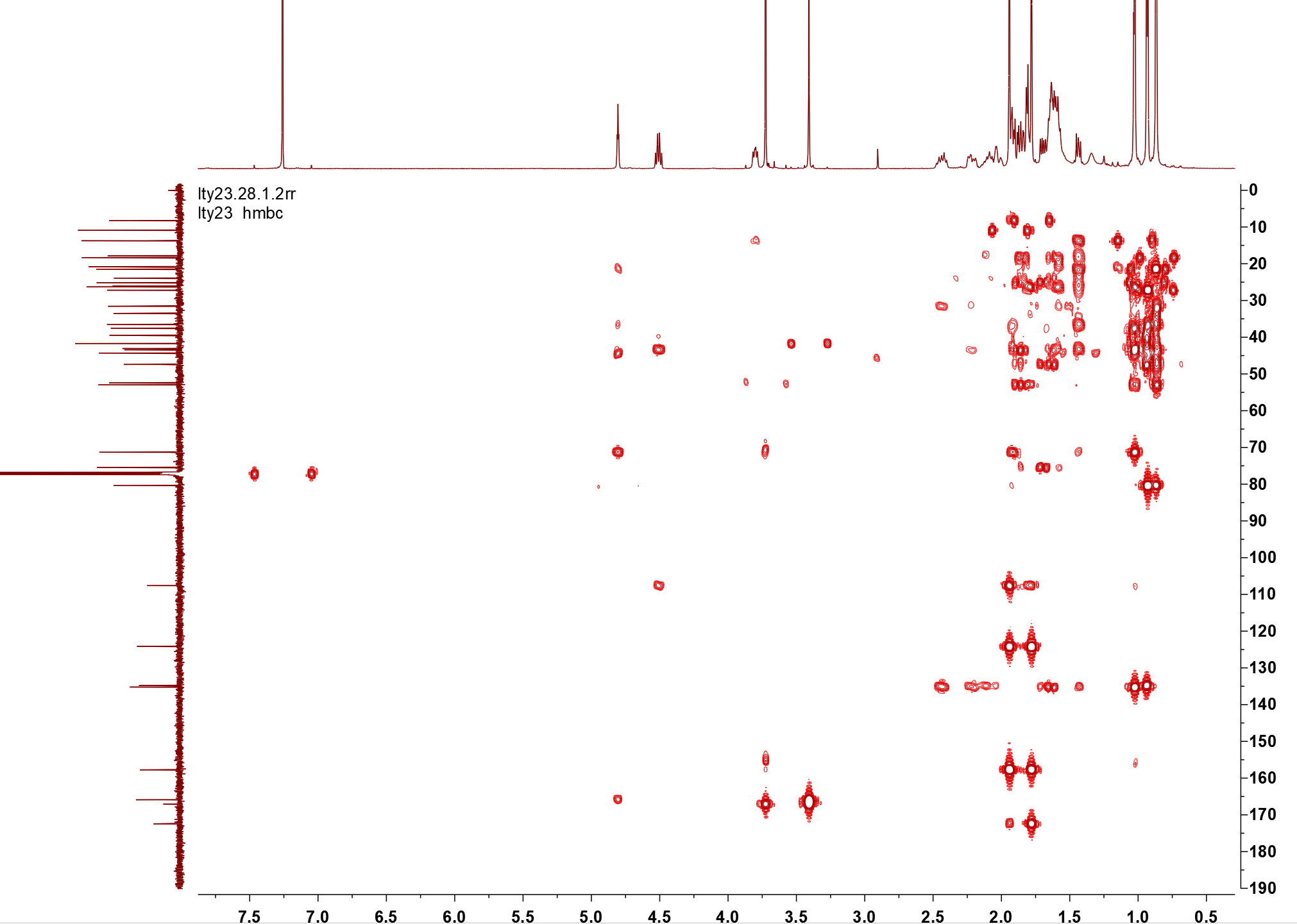


# Figure 15S. ROESY spectrum of **2**.


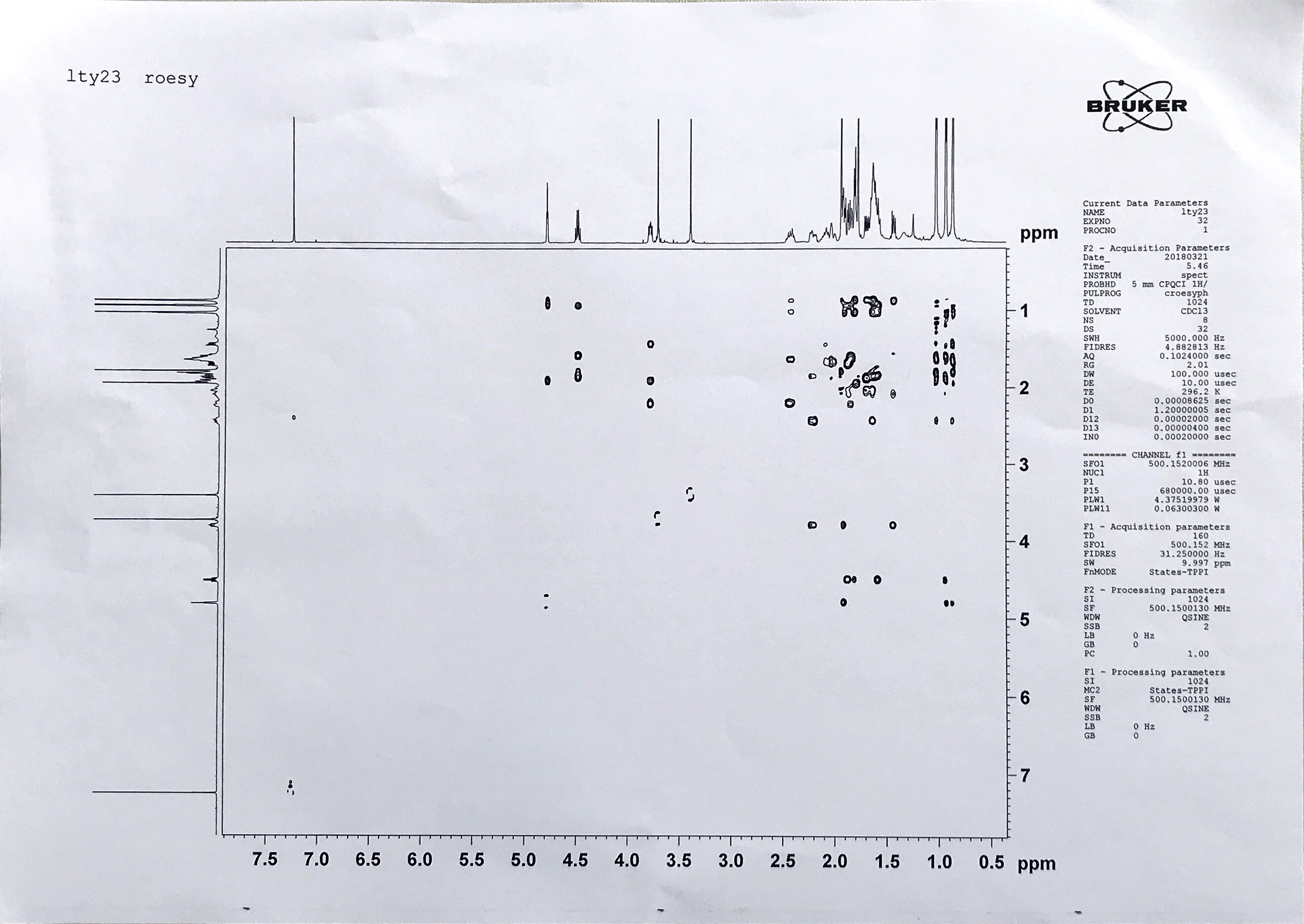


# Figure 16S. (+)-HRESIMS report of **2**.


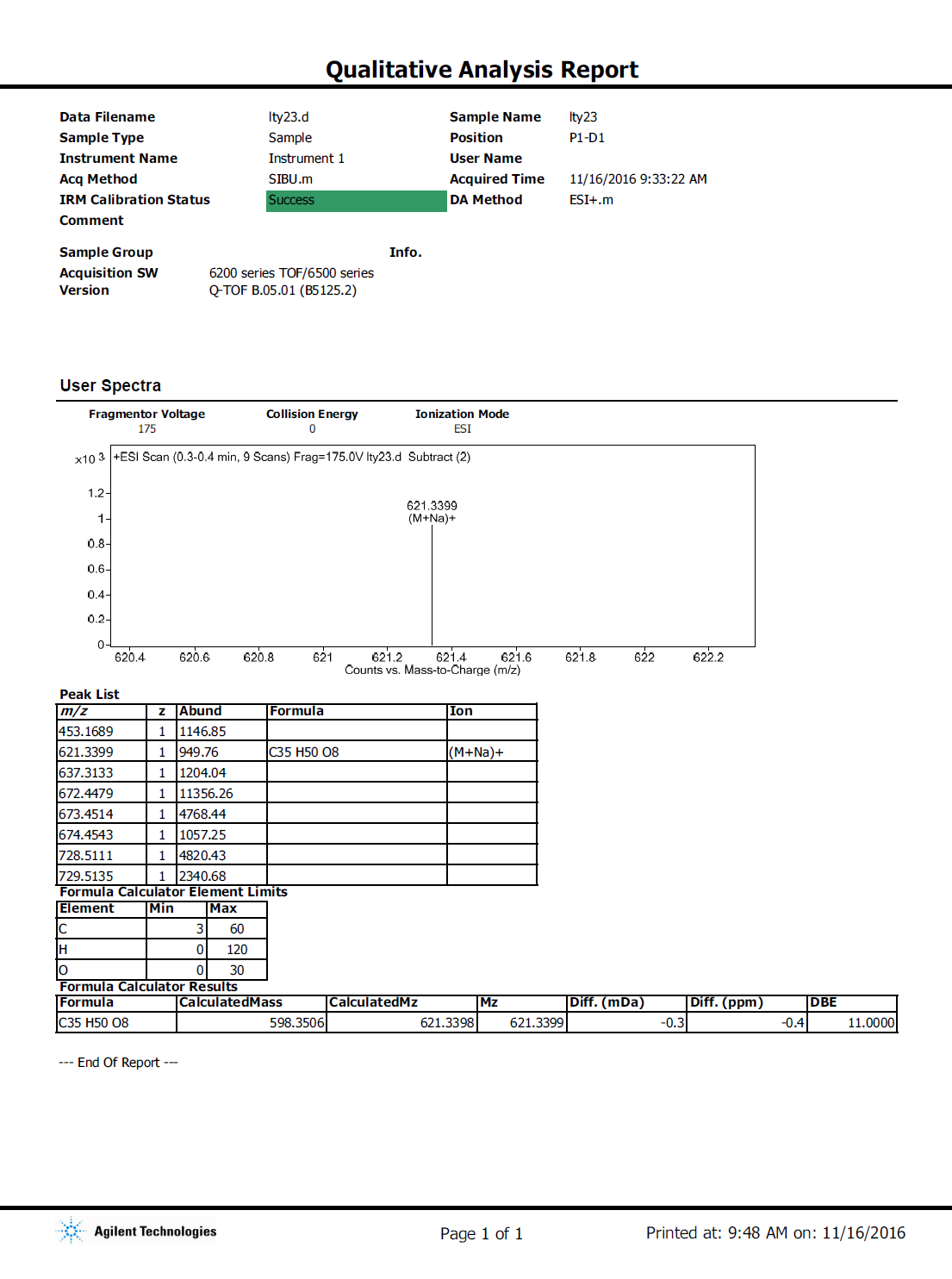


# Figure 17S. IR spectrum of **2**.


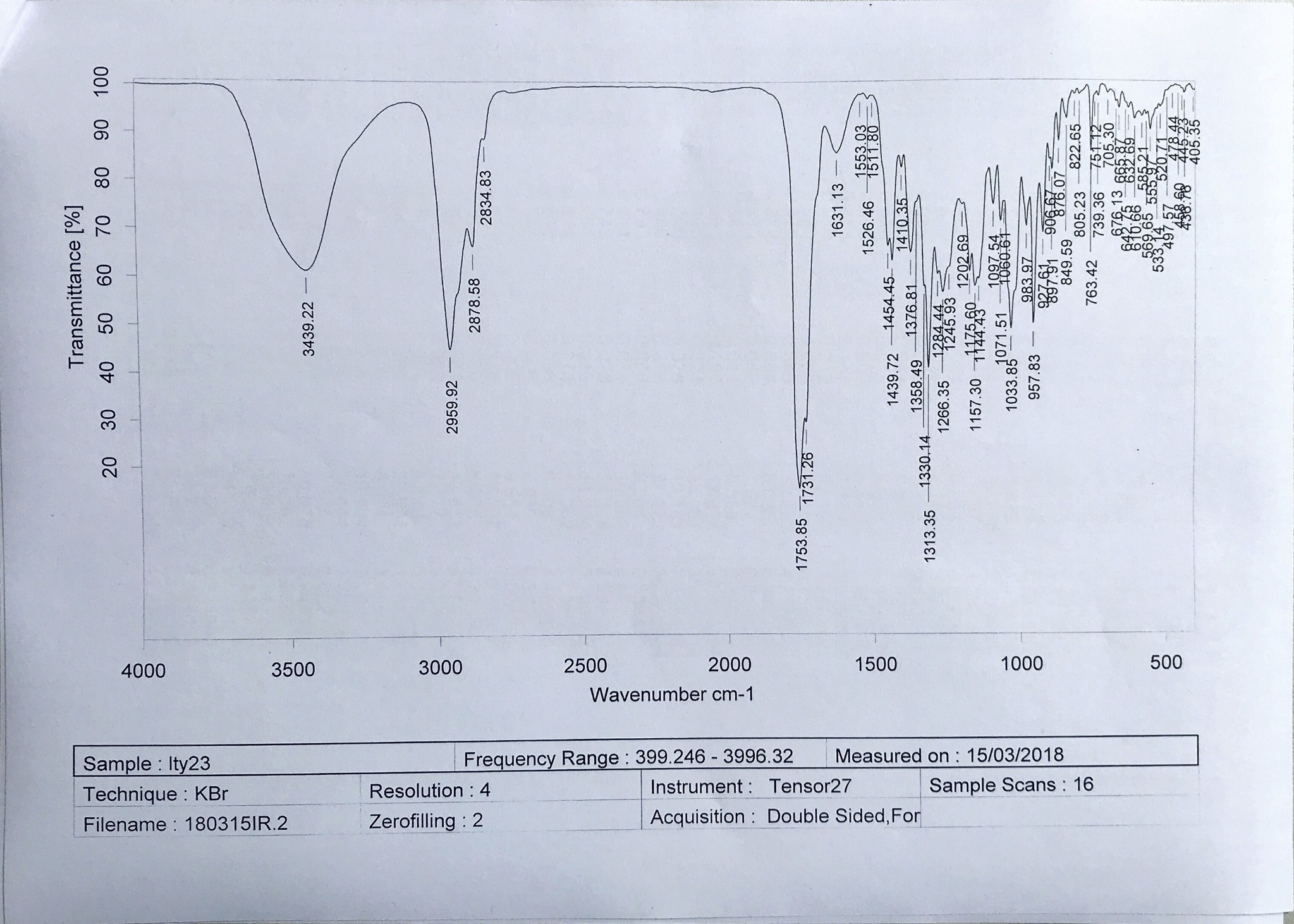


# Figure 18S. ^1^H NMR spectrum of **3** (500 MHz, CDCl_3_).


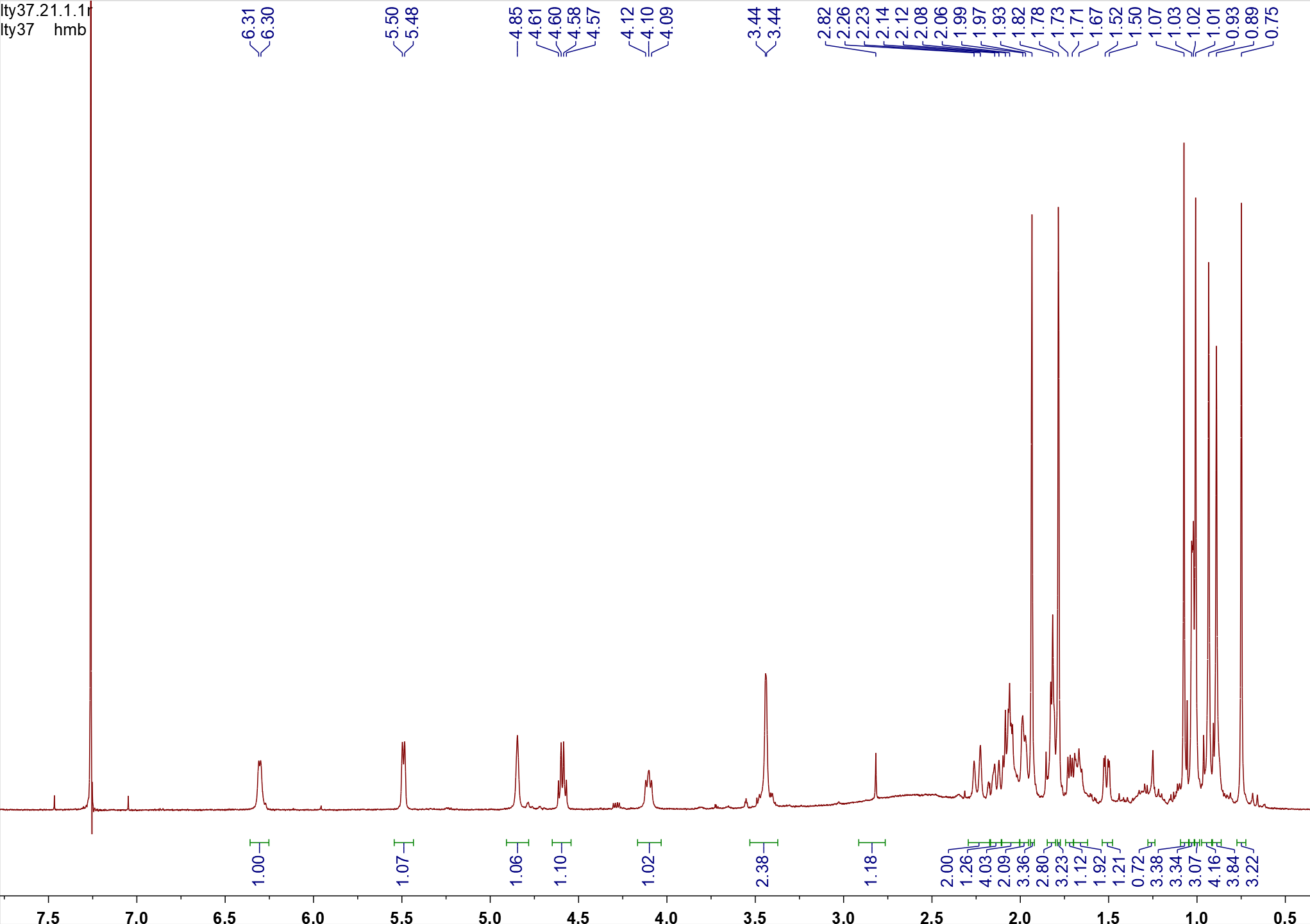


# Figure 19S. ^13^C NMR and DEPT spectra of **3** (125 MHz, CDCl_3_).


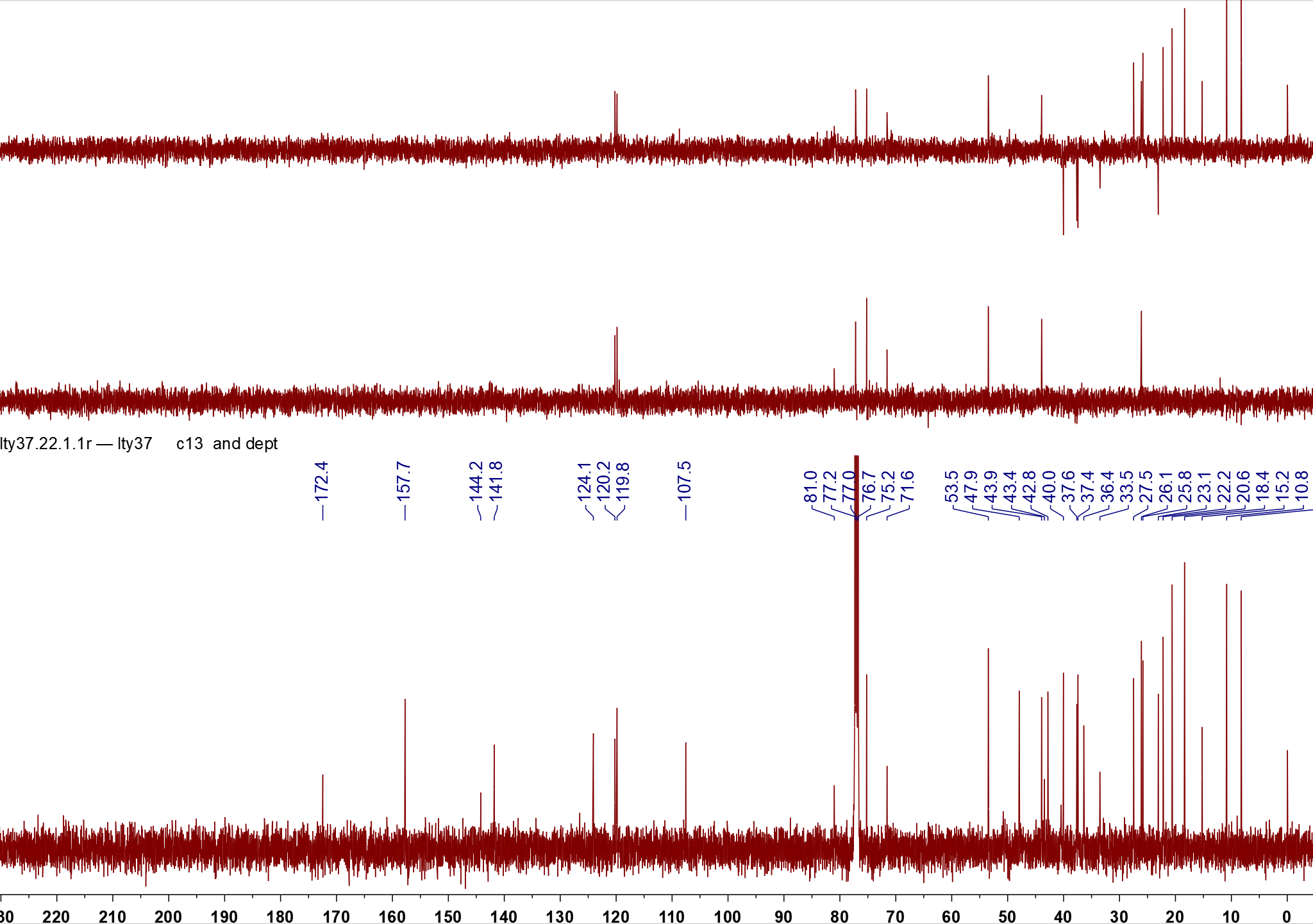


# Figure 20S. HSQC spectrum of **3**.


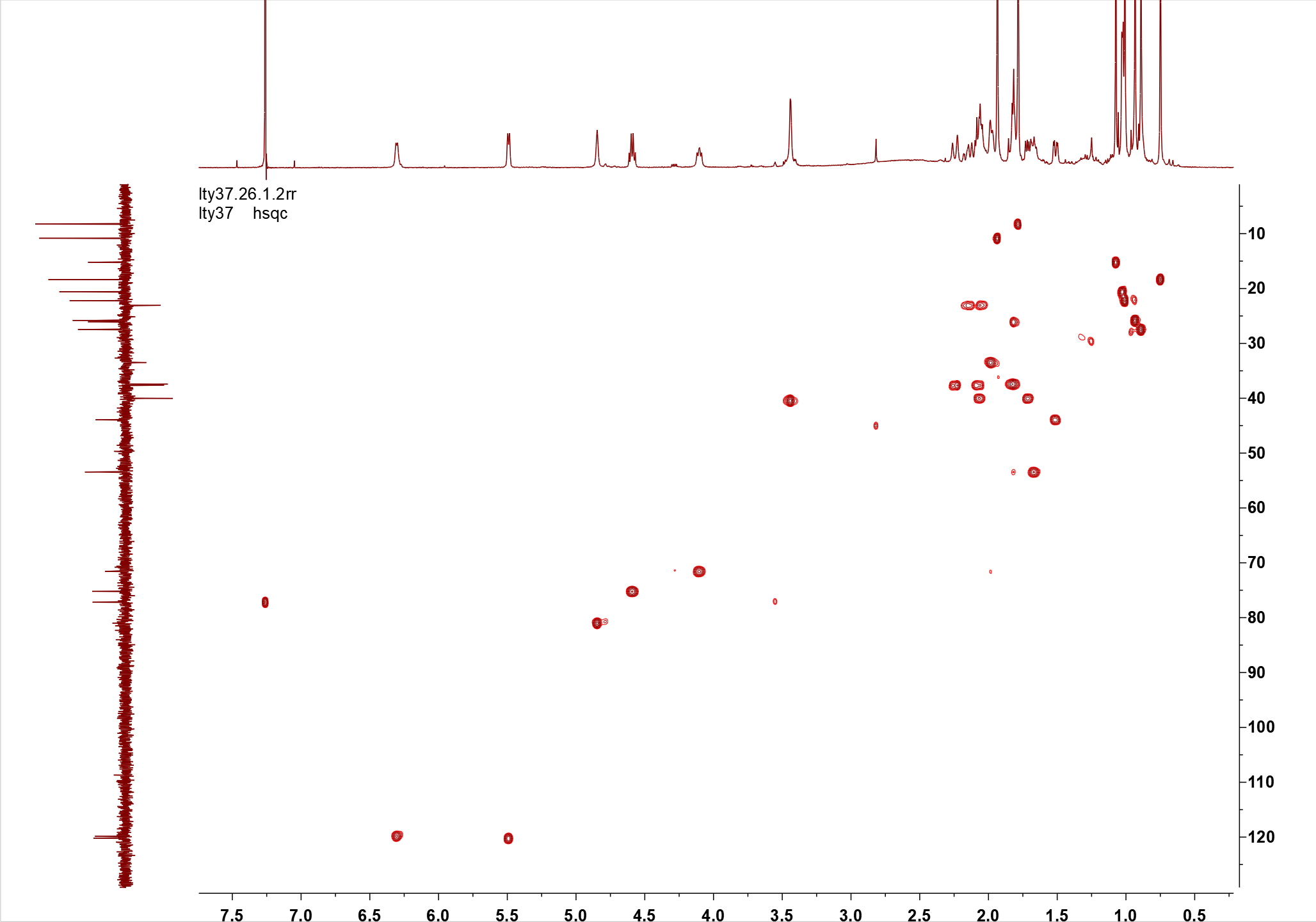


# Figure 21S. ^1^H-^1^H COSY spectrum of **3**.

# Figure 22S. HMBC spectrum of **3**.


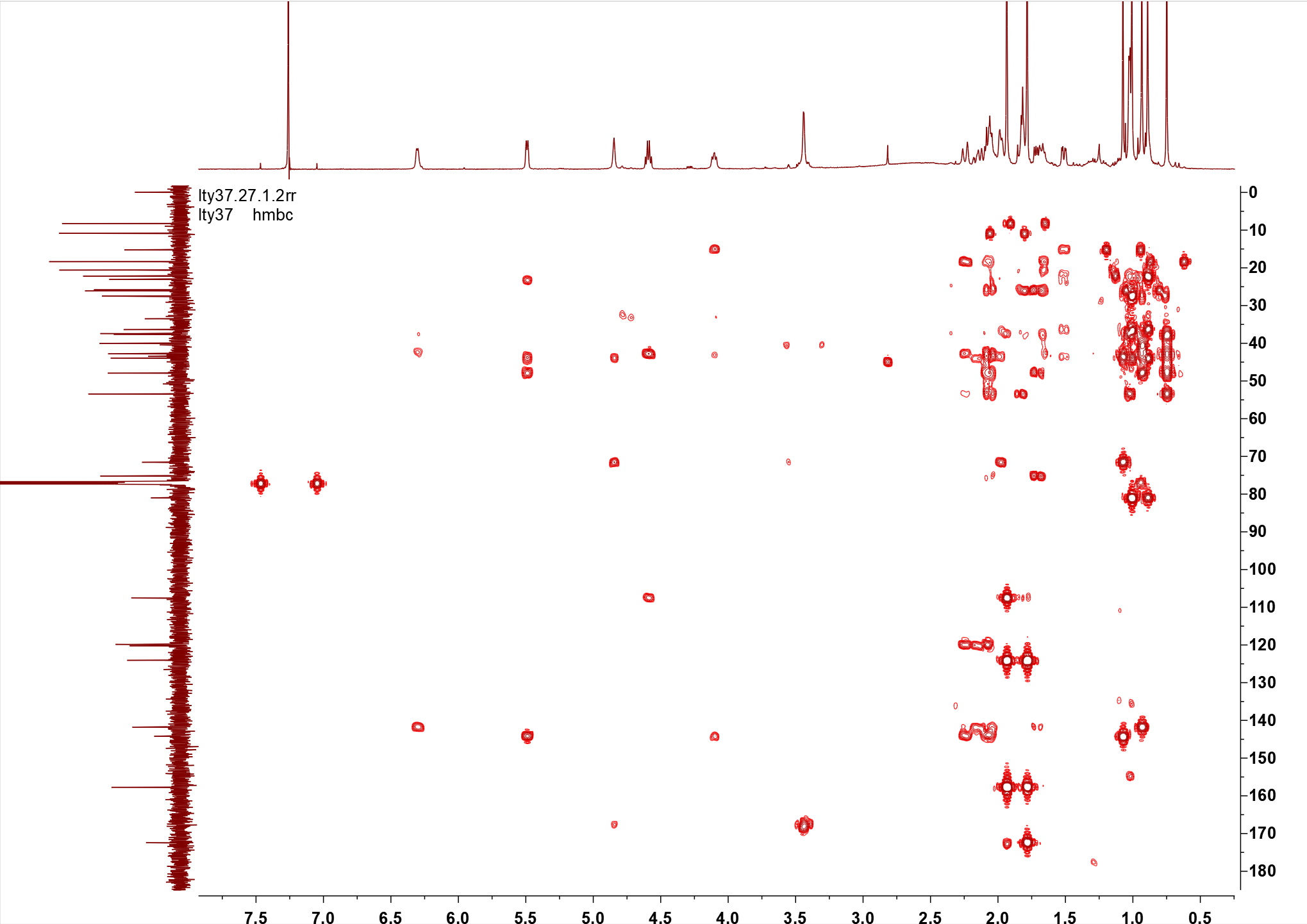


# Figure 23S. ROESY spectrum of **3**.


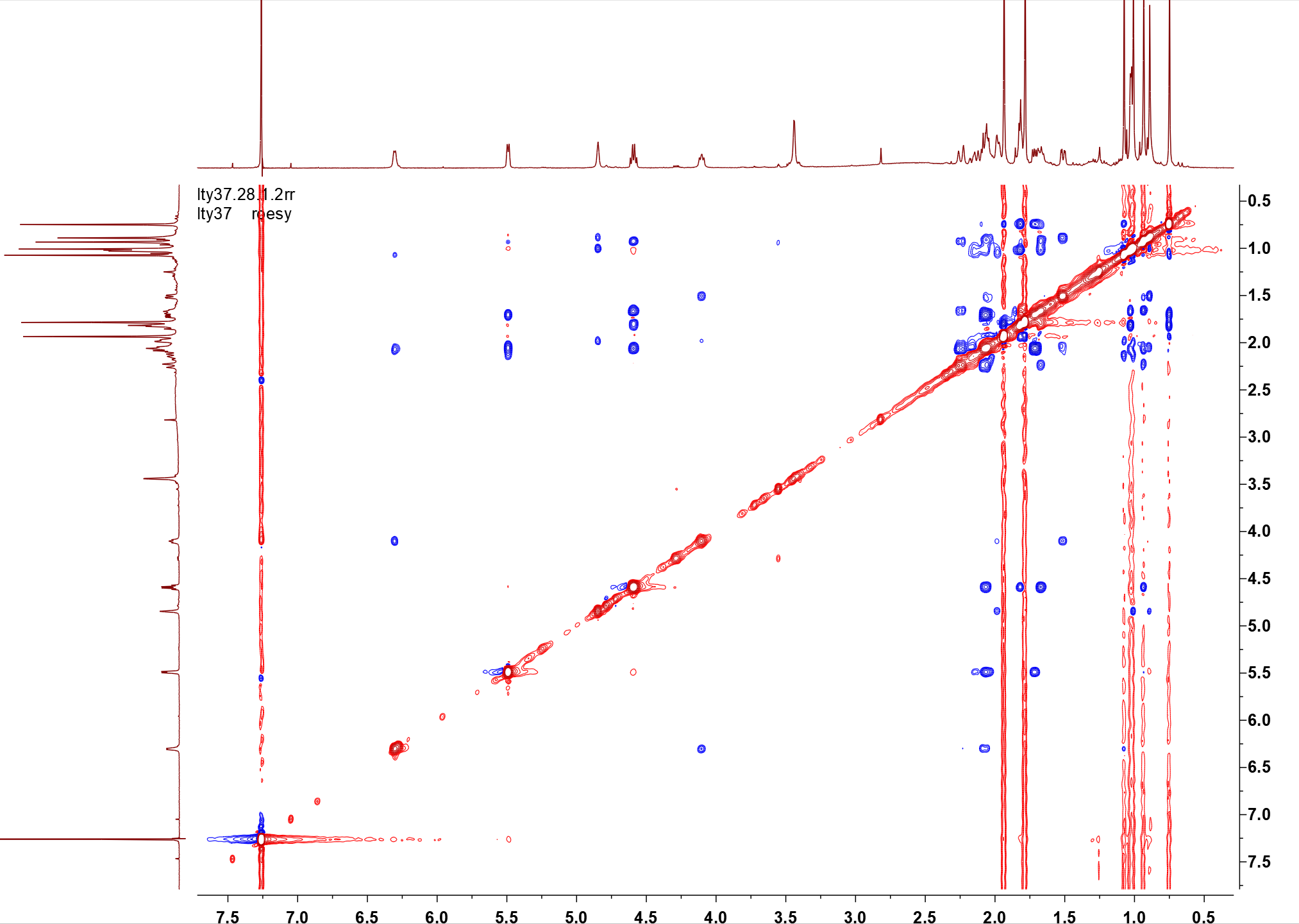


# Figure 24S. (+)-HRESIMS report of **3**.


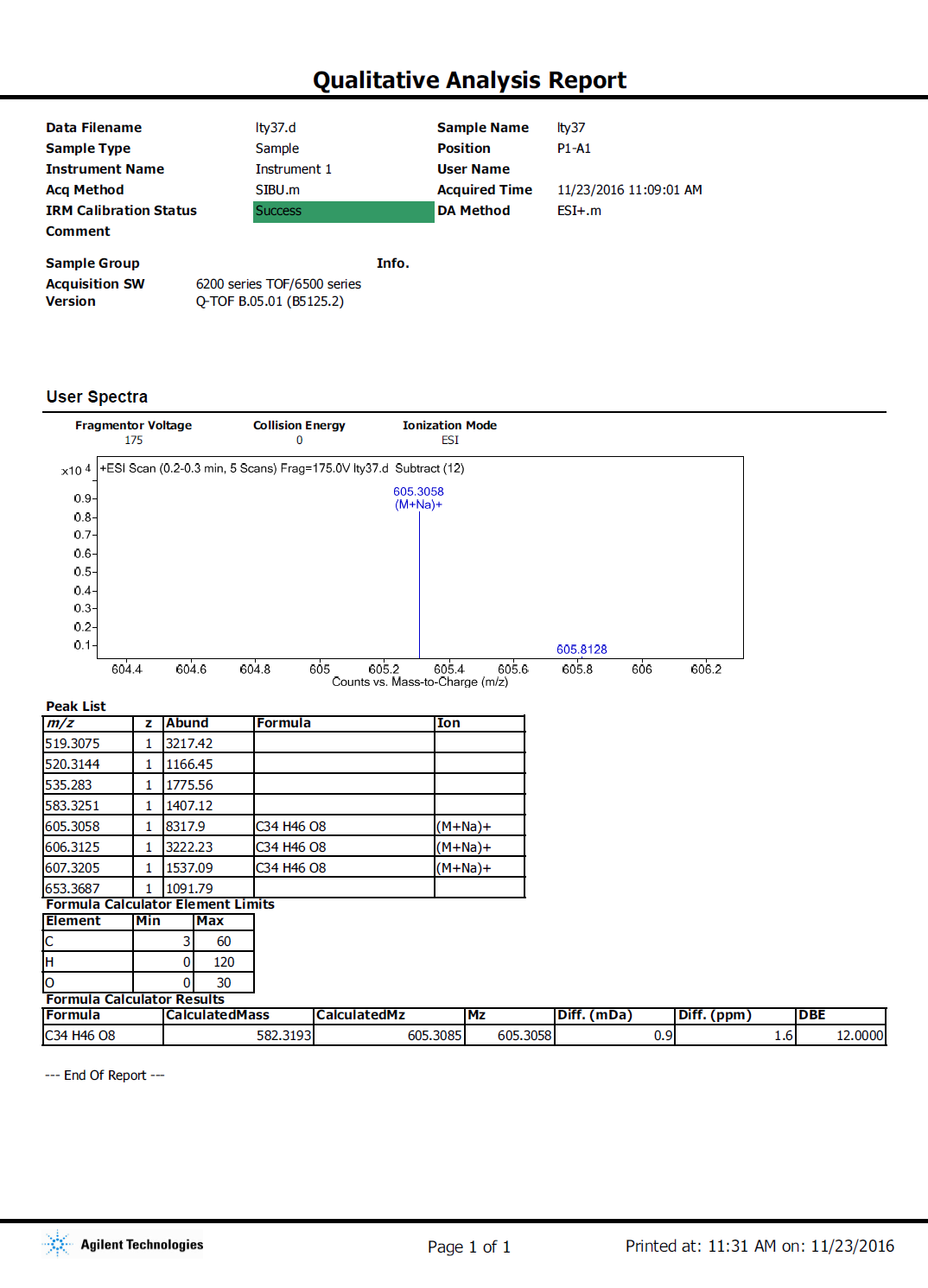


# Figure 25S. UV spectrum of **3**.


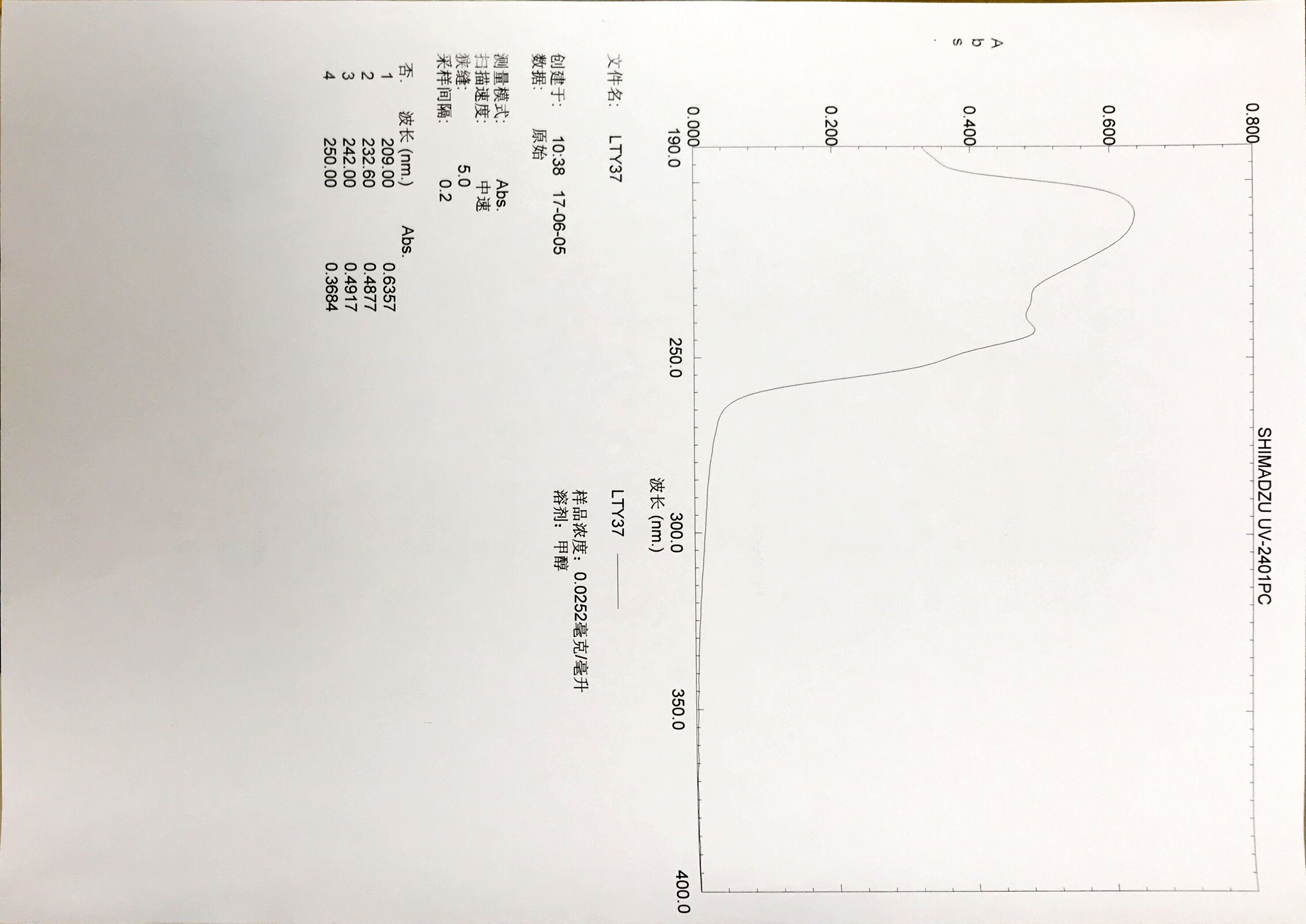


# Figure 26S. IR spectrum of **3**.


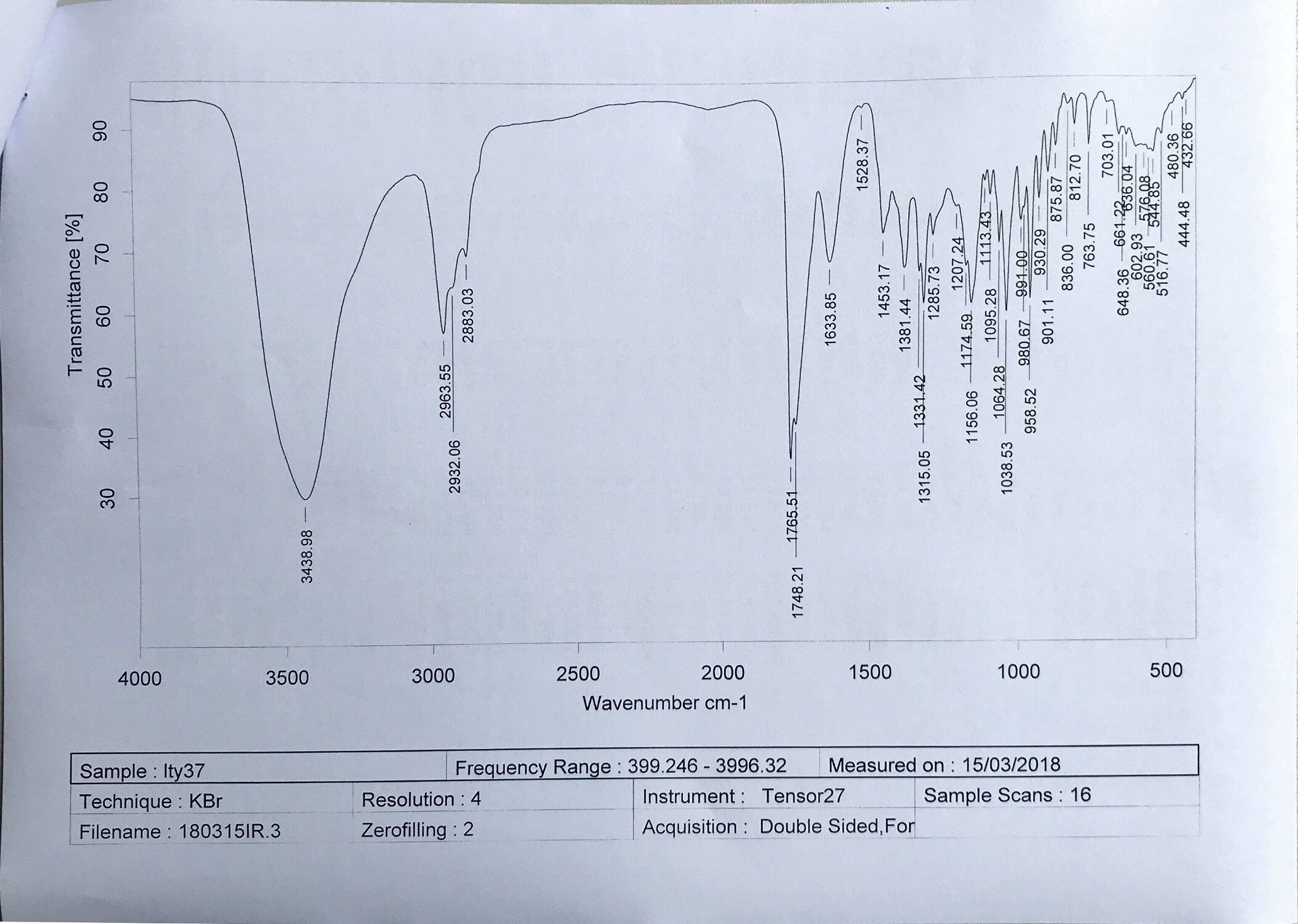


# Figure 27S. ^1^H NMR spectrum of **4** (600 MHz, CDCl_3_).


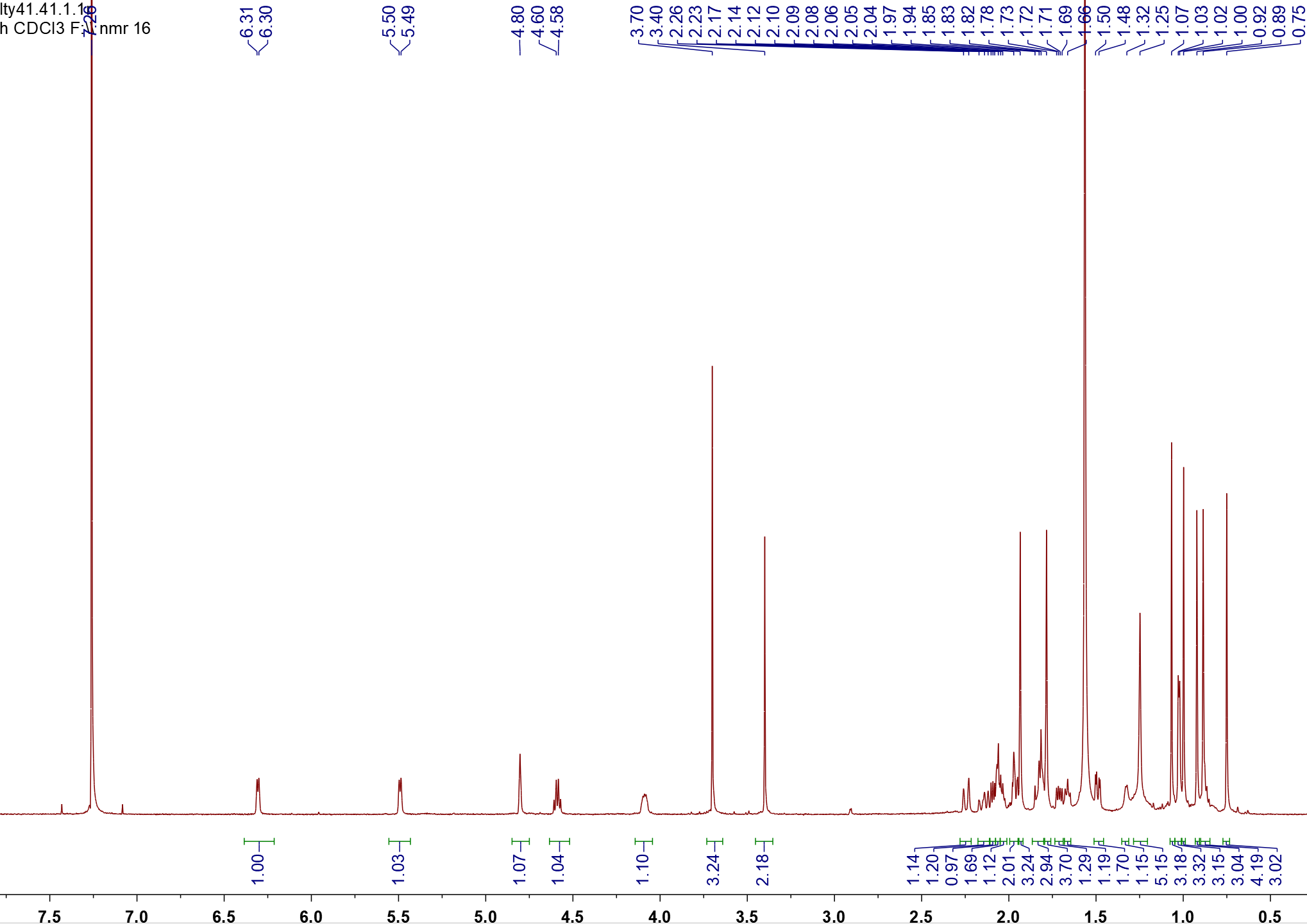


# Figure 28S. ^13^C NMR and DEPT spectra of **4** (150 MHz, CDCl_3_).


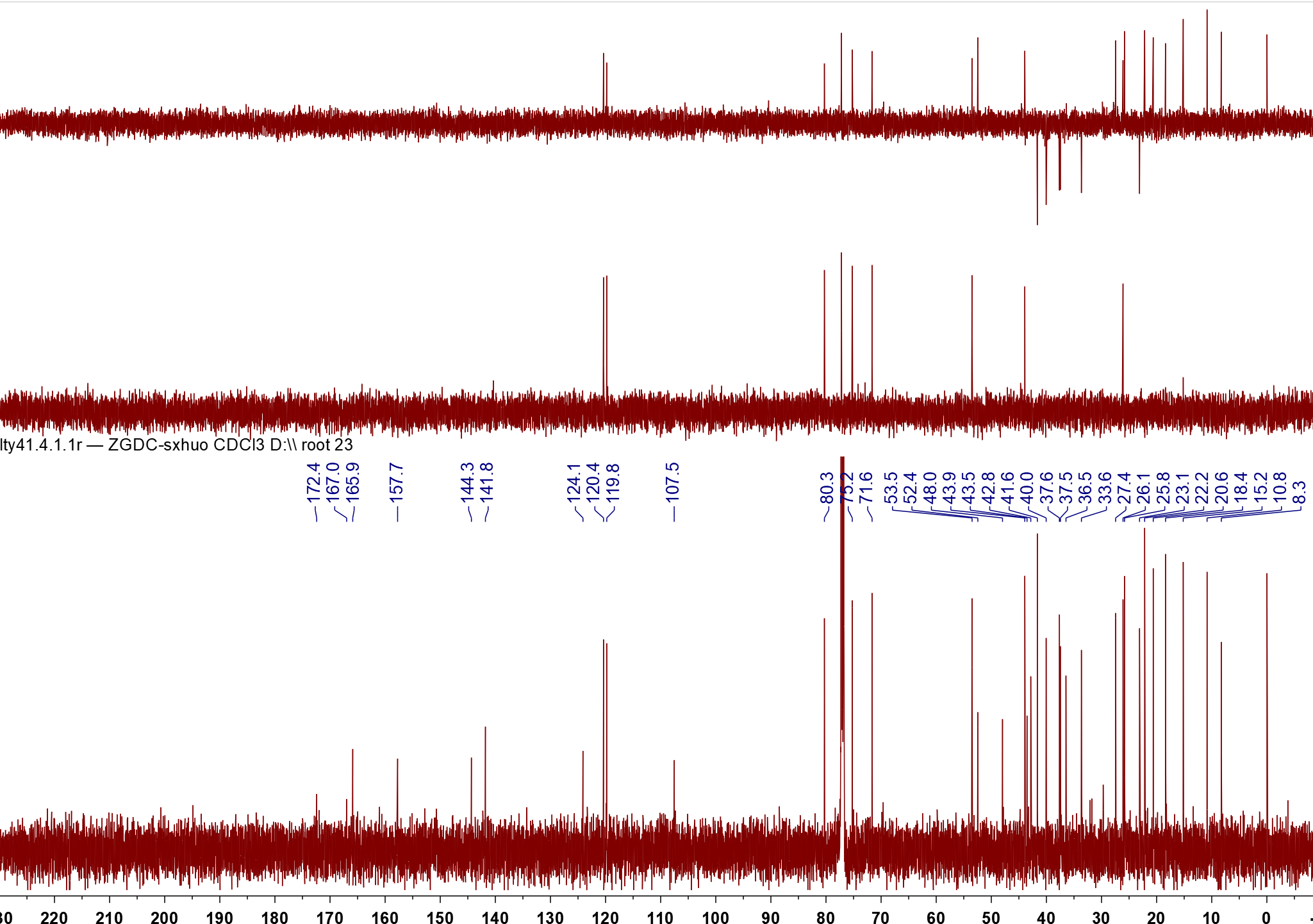


# Figure 29S. HSQC spectrum of **4**.


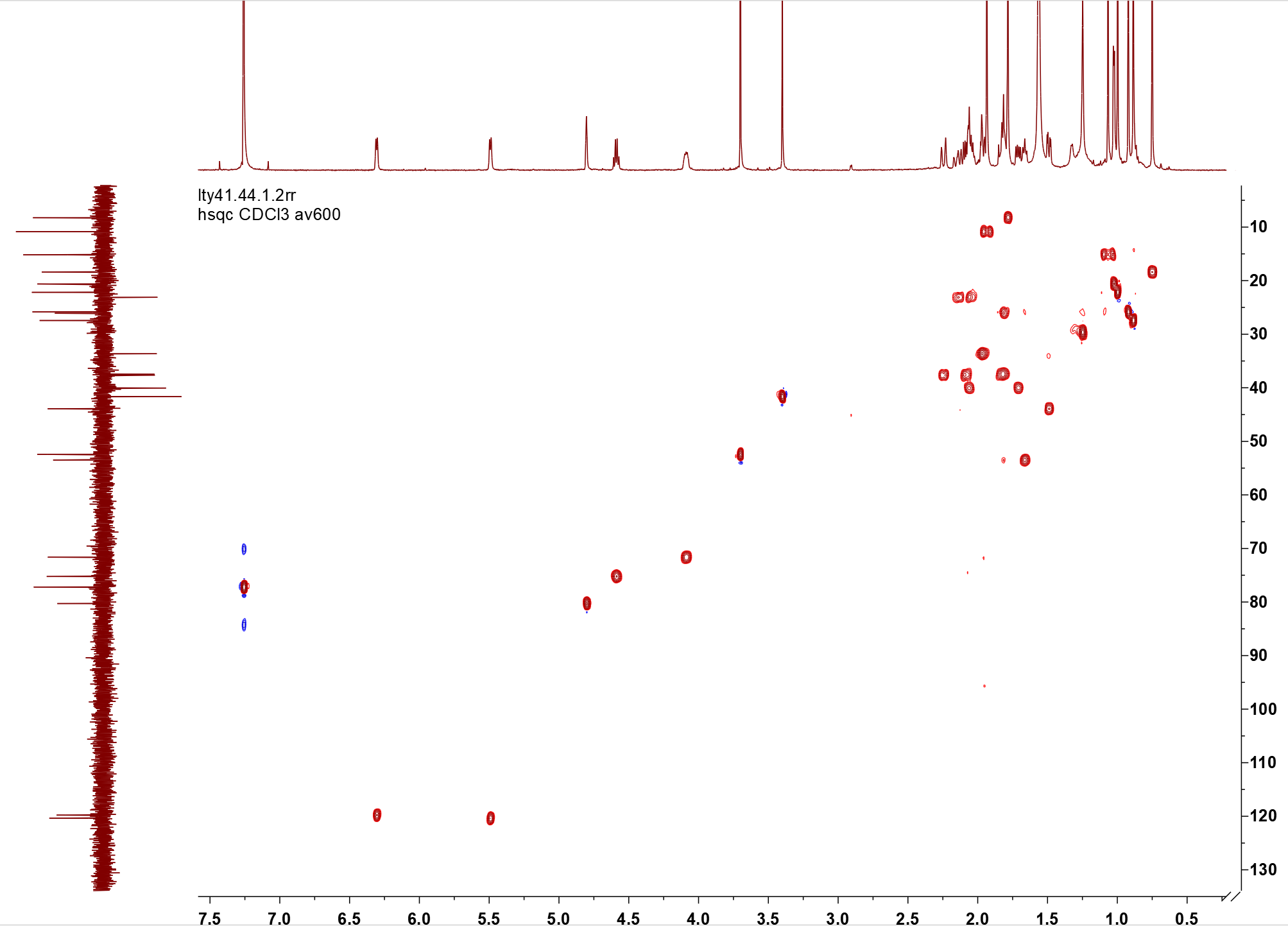


# Figure 30S. ^1^H-^1^H COSY spectrum of **4**.


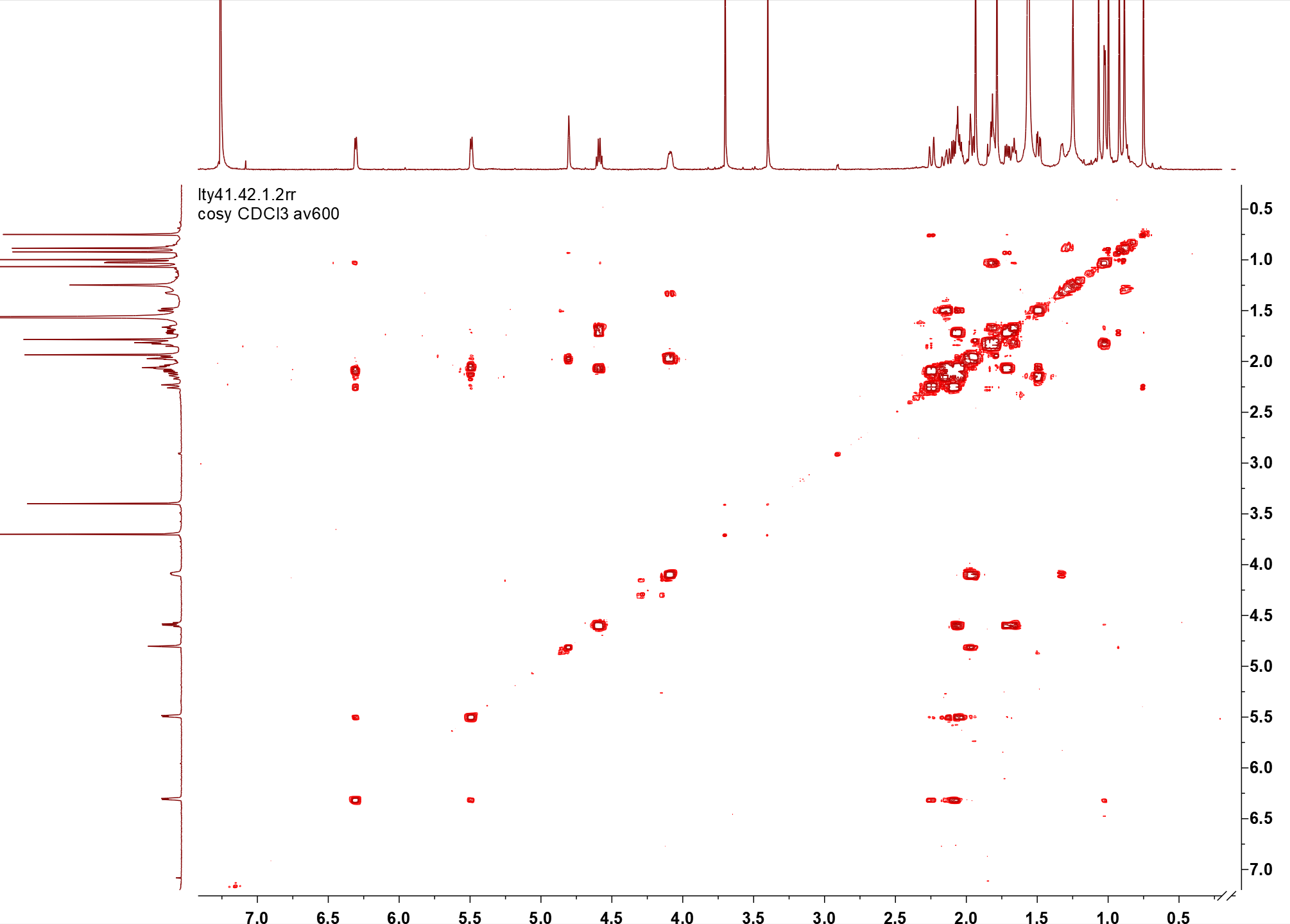


# Figure 31S. HMBC spectrum of **4**.


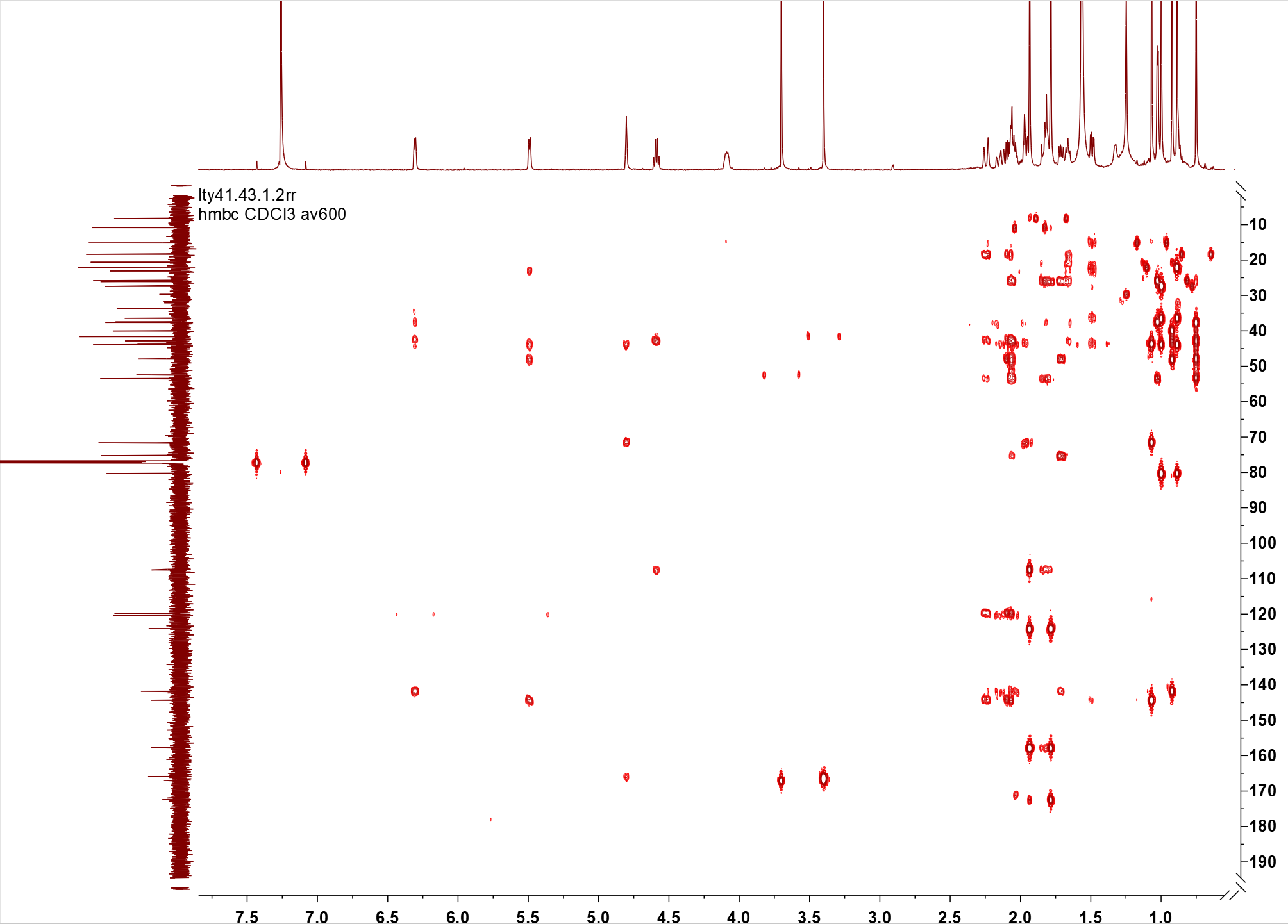


# Figure 32S. ROESY spectrum of **4**.


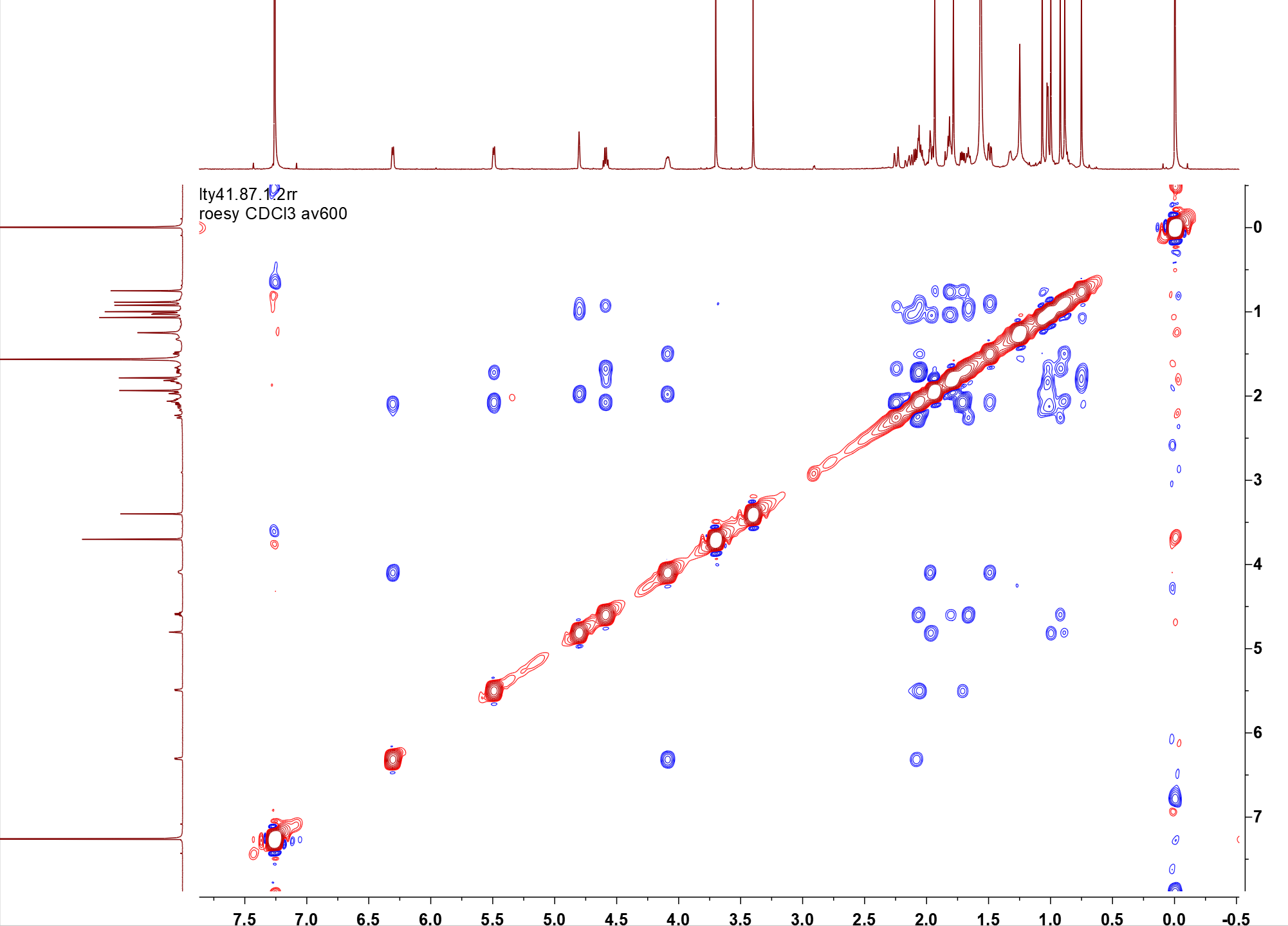


# Figure 33S. (+)-HRESIMS report of **4**.


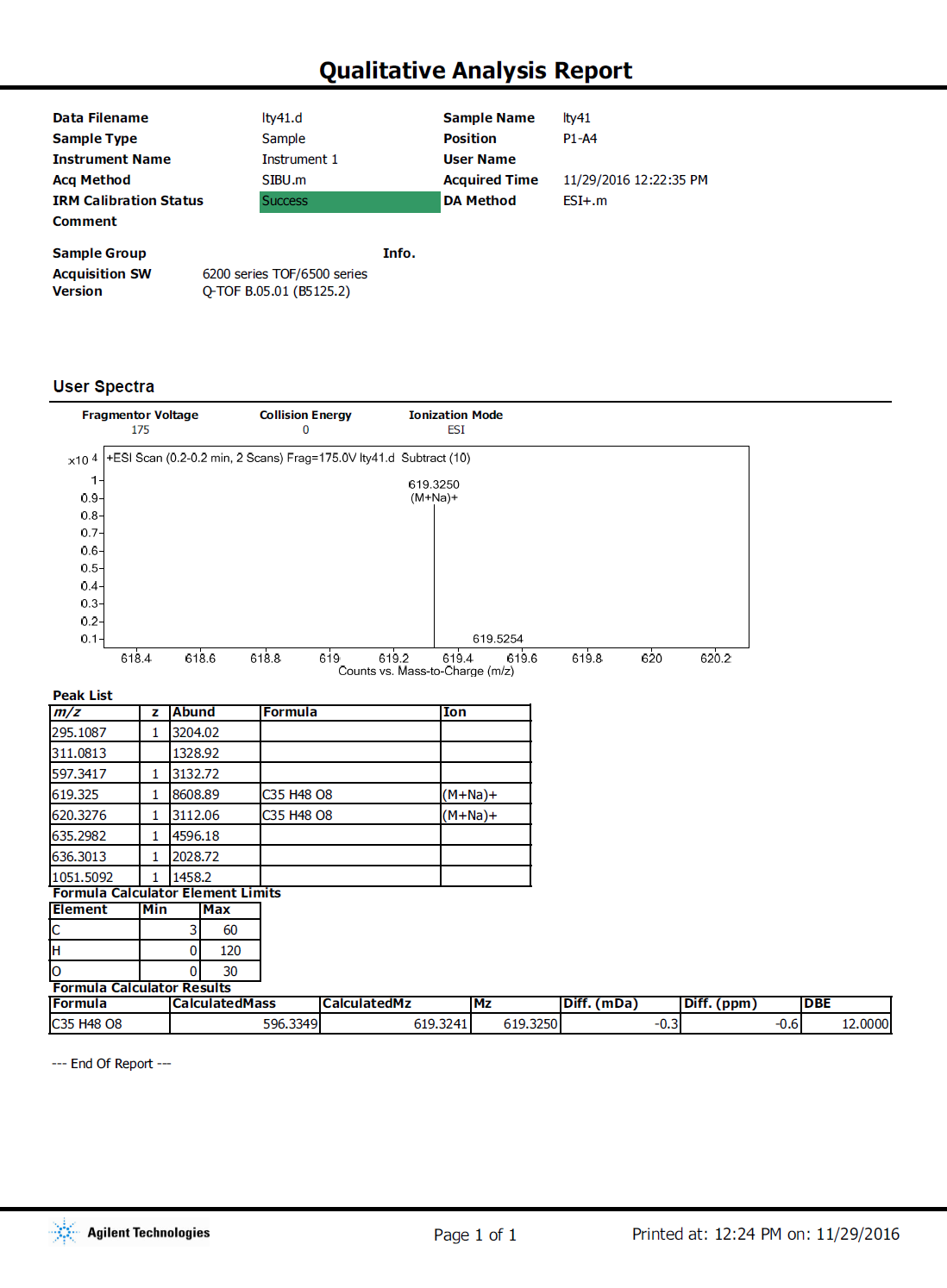


# Figure 34S. IR spectrum of **4**.


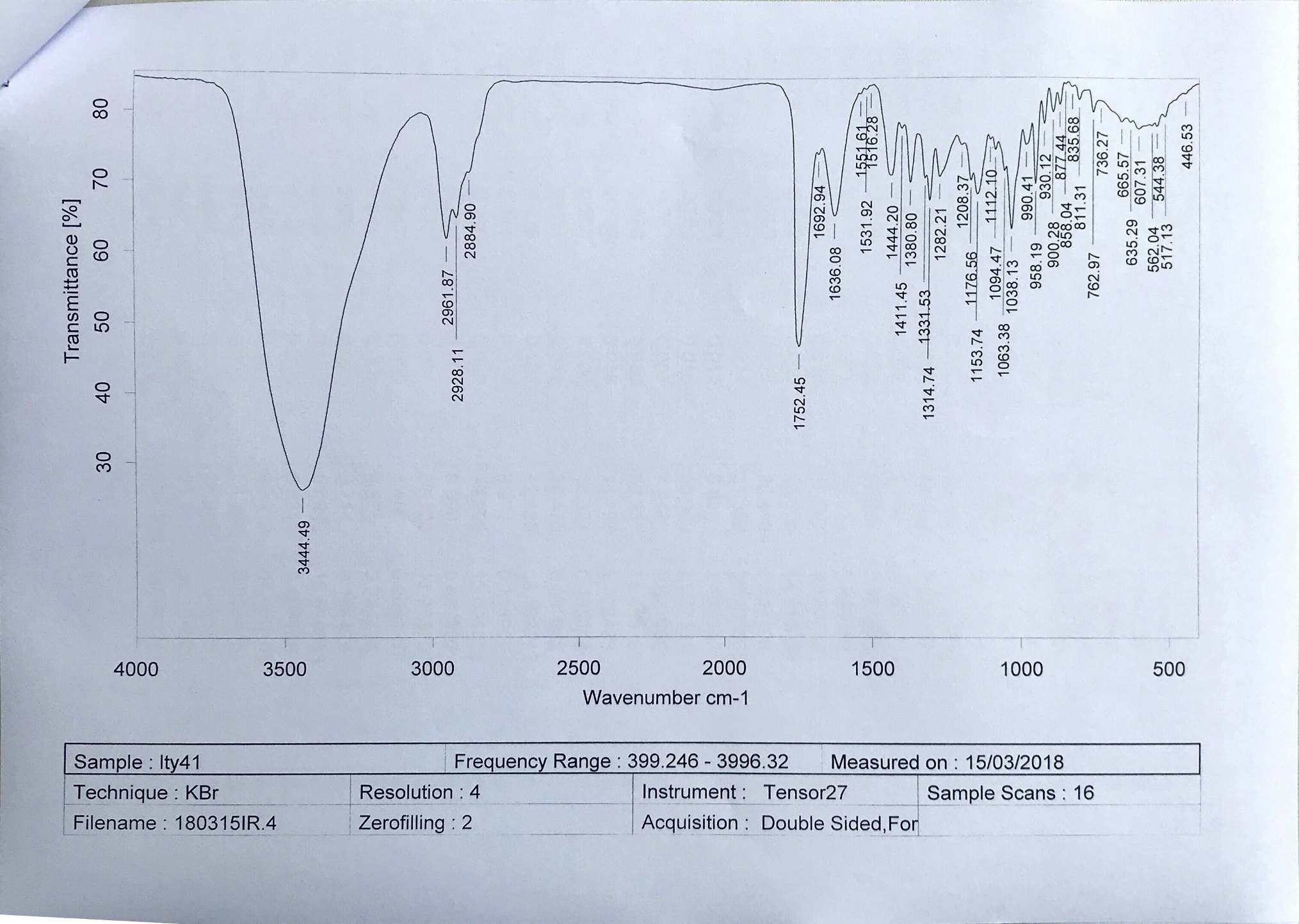

Supplement: Supplementary file 1 — Supplementary material 1 (DOCX 11171 kb) [file 13659_2018_160_MOESM1_ESM.docx]
